# Mitochondrial morphology in human fibroblasts and induced pluripotent stem cells in Leigh syndrome: A comparative analysis

**DOI:** 10.14814/phy2.70911

**Published:** 2026-05-10

**Authors:** Fibi Meshrkey, Ajibola B. Bakare, Raj R. Rao, Shilpa Iyer

**Affiliations:** ^1^ Department of Biological Sciences, J. William Fulbright College of Arts and Sciences University of Arkansas Fayetteville Arkansas USA; ^2^ Cell and Molecular Biology Program University of Arkansas Fayetteville Arkansas USA; ^3^ Department of Histology and Cell Biology, Faculty of Medicine Alexandria University Alexandria Egypt; ^4^ Department of Surgery, College of Medicine University of Arkansas Medical Sciences Little Rock Arkansas USA; ^5^ Department of Biomedical Engineering, College of Engineering University of Arkansas Fayetteville Arkansas USA

**Keywords:** mitochondria, morphology, networks, stem cells, structure

## Abstract

Mitochondria are dynamic organelles that regulate several vital cellular functions in both health and disease. Accurately quantifying different mitochondrial shapes using simple, affordable techniques remains challenging. We have previously developed a Mitochondrial Cellular Phenotype (MitoCellPhe) tool to quantify 24 different mitochondrial shapes, enabling sensitive analysis and quantification of mitochondrial phenotype in health, under stress, and in diseased conditions. This approach permits us to study the morphological changes, if any, associated with perturbations in the mitochondrial genome and function that contribute to mitochondrial diseases like Leigh Syndrome (LS), a fatal pediatric neurodegenerative and muscular disorder represented with different clinical phenotypes in infancy. Using images generated from normal and diseased fibroblasts and human induced pluripotent stem cells (hiPSCs) (undifferentiated), we have identified and characterized differences in morphologies between a healthy and diseased state in both undifferentiated hiPSCs and differentiated fibroblasts. These results will help us better understand the pathophysiology of devastating mitochondrial diseases like LS, especially in its early developmental stages.

## 1 INTRODUCTION

Mitochondria are essential organelles leading multiple cellular functions, with a primary purpose of sustaining cellular energy demands via the production of a high‐energy molecule, adenosine triphosphate (ATP) (Duchen, 2004). Mitochondria are in a continuous dynamic state of fission and fusion (JG et al., 2013; Sprenger & Langer, 2019). The dynamic form in each cell varies with its metabolic needs and developmental stage. Variations in the mitochondrial phenotypes may lead to bioenergetics defects and vice versa (Westermann, 2012). Mitochondrial dysfunction is currently involved in the pathogenesis of several adult human diseases, such as neurodegeneration (Gao et al., 2017), and cancer (Duchen, 2004; Wallace, 2012).

Primary mitochondrial disorders, such as Leigh syndrome (LS), are characterized by early onset of neurological symptoms and myopathy in children (Bakare, Lesnefsky, & Iyer, 2021). Further, LS can also be caused either due to a nuclear DNA mutation or maternally transmitted due to a mitochondrial DNA (mtDNA) mutation (Bakare, Lesnefsky, & Iyer, 2021). These mutations usually affect the oxidative phosphorylation complexes present in the inner mitochondrial membrane and are responsible for the majority of ATP production in the cell (Bakare, Dean, et al., 2021; Bakare, Lesnefsky, & Iyer, 2021). Nevertheless, it is unclear whether mitochondrial morphological changes associated with bioenergetic defects are correlative or causative. Due to this critical but elusive association, several studies have focused on understanding the relationship between the morphology and function of the mitochondria in health and disease (Bakare, Daniel, et al., 2021; Gao et al., 2017; Meshrkey et al., 2021). Quantifying mitochondrial morphologies associated with mtDNA mutations in disease is challenging because of the number of mitochondria and the heterogeneity of mitochondrial networks with varying branch lengths in a single cell. Therefore, precisely mapping different mitochondrial morphologies associated with mitochondrial diseases could aid in a critical understanding of the pathogenesis of these diseases and help in developing new therapeutic protocols specific to each disease based on mitochondrial morphology.

Understanding the exact mitochondrial phenotype changes (if any) and their relation to mtDNA mutation in early development requires further investigation, meticulously mapping the different mitochondrial morphologies within a single cell. In this study, we have used the newly developed mitochondrial phenotype analysis tool (MitoCellPhe) (Bakare, Meshrkey, et al., 2021), to measure the mitochondrial structure of reprogrammed human induced pluripotent stem cells (hiPSCs) from their parental fibroblast cell lines from four LS patients containing mtDNA mutations impacting Complex I and Complex V subunits of the respiratory chain (Grace et al., 2019). The diseased cell lines used in this study carry some of the most prevalent mutations involved in LS (Bakare, Dean, et al., 2021). Live cells stained with Mitotracker Red (MTR) were analyzed to map the various mitochondrial morphologies in health and disease compared to healthy controls.

## 2 MATERIALS AND METHODS

### 2.1 Cell culture

Cultures of human BJ (ATCC® CRL‐2522™) fibroblasts were obtained from the American Type Culture Collection (ATCC, Manassas, VA). The SBG2 (*m.T8993G*), SBG3 (*m.T9185C*), SBG4 (*m.T10158C*), and SBG5 (*m.T12706C*) diseased fibroblasts were obtained from the Medical University of Salzburg, Austria (Bakare, Dean, et al., 2021). These cells were maintained in a fibroblast expansion medium consisting of minimal essential medium (MEM) (Catalog #10370088; Thermo Fisher Scientific, Waltham, MA, USA), 10% fetal bovine serum (FBS) (Catalog #SH30071.03; GE Healthcare‐HyClone™, Chicago, IL, USA), and 2 mM L‐glutamine (Catalog #25030081; Thermo Fisher Scientific, Waltham, MA, USA). Fibroblasts were enzymatically passaged in 0.05% Trypsin‐EDTA (Catalog #2530054; Thermo Fisher Scientific, Waltham, MA, USA).

Reprogrammed hiPSCs from the fibroblasts were regularly maintained in NutriStem hPSC xeno‐free (XF) medium (Catalog #05‐100‐1A; Reprocell USA Inc., Beltsville, MD, USA) with Stemolecule Y27632 Dihydrochloride Hydrate (Catalog #04‐0012‐02; Reprocell USA Inc., Beltsville, MD, USA) in a laminin‐511 E8 fragment matrix, iMatrix 511 (Catalog #NP892‐012; Reprocell USA Inc., Beltsville, MD, USA) coated dishes. Medium was changed daily. hiPSCs were enzymatically passaged once reaching 70%–80% confluency using StemPro Accutase (Catalog #A11105‐01; Thermo Fisher Scientific, Waltham, MA, USA) (Grace et al., 2019; Meshrkey et al., 2021, 2023).

All fibroblast and hiPSC cell cultures were maintained without the use of antibiotics, handled in Biosafety Type II sterile hoods regularly cleaned with UV irradiation and 70% ethanol, and grown in 37°C incubators at 5% CO_2_ and 95% humidity. When the cells were 70% confluent, they were passaged for fluorescence labeling and image analysis, as detailed in the next section. Before use in experiments detailed below, cells were dissociated using 0.05% trypsin–EDTA (Catalog #2530054; Thermo Fisher Scientific, Waltham, MA, USA). The fluorescence labeling and image analysis were performed on passage 9 for fibroblasts, and 20,000 cells were seeded into 35 mm dishes, while hiPSCs were seeded at 10^5^ cells per 35 mm dish and imaged at passage 11 in culture.

### 2.2 Fluorescence labeling of mitochondria in fibroblasts and hiPSCs

To label the mitochondria, fibroblast cells were incubated with MEM NEAA basal medium without L‐glutamine (Catalog #10370021; Thermo Fisher Scientific, Waltham, MA, USA) containing 150 nM Mitotracker Red CM‐H2Xros (Catalog #M7513; Thermo Fisher Scientific, Waltham, MA, USA) for 30 min. In the FCCP (trifluoromethoxy carbonyl cyanide phenylhydrazone) (Catalog #C2920; MilliporeSigma, Rockville, MD, USA) treatment group, fibroblast cells were incubated with 0.7 μM FCCP for 30 min before the addition of the Mitotracker Red CM‐H2Xros. At the end of the incubation period, the cells were washed three times with pre‐warmed Dulbecco's phosphate‐buffered saline (dPBS). The nucleus was stained by incubating cells in a basal medium containing Nucblue Hoechst 33342 (Catalog # R37605; Thermo Fisher Scientific, Waltham, MA, USA) for 15 min. Following this incubation, cells were washed several times with pre‐warmed dPBS to remove excess dye. At the end of the wash, MEM NEAA basal medium was added to each dish before image acquisition.

To visualize mitochondrial morphology in the hiPSCs, cells were seeded in a 35 mm pre‐coated culture dish. The cells were incubated for at least 48 h before staining. Subsequently, a 100 nM solution of Mitotracker Red CMXRos (Catalog # M5712; Thermo Fisher Scientific, Waltham, MA, USA) prepared in a serum‐free culture medium was added to stain the cells maintained in a 37°C incubator at 5% CO_2_ and 95% humidity. At the end of the incubation period, the cells were washed 3 times with pre‐warmed Dulbecco's phosphate‐buffered saline (dPBS) (Catalog #21‐040‐CVR; MilliporeSigma, Rockville, MD, USA). The nucleus was stained by further incubating cells with a basal medium containing Nucblue Hoechst 33342 for 15 min. Following this incubation, cells were washed several times with pre‐warmed dPBS to remove excess dye. At the end of the wash, MEM NEAA basal medium was added to each dish before image acquisition.

### 2.3 Live‐cell fluorescence microscopy

Fluorescence images of live cells were acquired using an EVOS FL inverted light/epifluorescence microscope with a 40×/0.65 objective and a Sony ICX445 monochrome CCD digital camera. Red fluorescence from Mitotracker Red was measured using a 530 nm excitation and a 593 nm emission filter set. Blue fluorescence from Nucblue Hoechst was measured using a 360 nm excitation and a 447 nm emission filter set. All live cells were imaged on 35 mm dishes containing phenol‐red‐free basal medium. Image acquisition was performed one dish at a time with a maximum time of 30 min between dishes. Images were acquired using the EVOS XL Cell Imaging System (version #26059; Thermo Fisher Scientific, Waltham, MA, USA). All dishes were stored in a humidified 37°C, 5% CO_2_ incubator until image acquisition. All images of live cells were taken on the same day as the labeling of mitochondria. All live‐cell images were exported as TIFF files for further analysis. Five to 10 images were acquired per dish, and three dishes were stained per trial. At least three independent trials were performed for both the fibroblasts and hiPSC lines.

### 2.4 Mitochondrial phenotype analysis with MitoCellPhe

MitoCellPhe Skeletonizer and Analyzer were used to analyze the images. The underlying algorithms for the MitoCellPhe skeletonizer and Analyzer have been previously discussed extensively (Bakare, Meshrkey, et al., 2021). To enable analysis of a raw cell image, the image must first be skeletonized. Skeletonization allows binary images to be reduced to 1‐pixel width. Additionally, if the goal of the analysis is to evaluate individual cells, the image must be segmented further into regions that define individual cells. Before skeletonizing a cell image, the image must be pre‐processed to improve the quality of the skeleton.

The image pre‐processing consists of converting the stained sample image to skeletonized images for each cell detected. The input image is a three‐channel image displaying the applied red mitochondrial stain. To perform the morphology analysis, a binary mitochondrion skeleton image representing the stained sample image is required. The grayscale mitochondria images contain significant variation in pixel intensity across regions of the image. Therefore, to prepare the stained image for skeletonization, we used Contrast Limited Adaptive Histogram Equalization (CLAHE) illumination correction (Zuiderveld, 1994) with a 10 × 10 kernel size, a clip limit of 0.02, and 256 bins. The small kernel size helps to even the brightness of the mitochondria. To generate the binary image for skeletonization, the CLAHE output is thresholded using a 2‐class adaptive Otsu thresholding method. This method works well on stained images that have been CLAHE normalized (Otsu, 1979). Afterward, the morphological skeleton can be generated.

The Otsu‐thresholded image is then used as the start image for the skeletonization process. The skeletonization of the image is accomplished with Zhang's method (Zhang & Suen, 1984), which performs successive passes over the thresholded image to detect boundary pixels and whittle them down to one‐pixel‐wide branches. The segmentation process, if desired, can split the skeletonized image into separate regions for each cell in the image.

If the stained sample image contains multiple cells, and they are not clustered close together (as is the case with the hiPSC lines), then MitoCellPhe can additionally segment the sample image into multiple images of each of its cells. Such cell types are called differentiated cell types, and those that are clustered are called undifferentiated cell types. For undifferentiated cell types, an aggregate morphology analysis can be performed without using segmentation. When running in MitoCellPhe Analyzer, the tool will perform morphology analysis on the entire image. For differentiated cell types, the images can be used directly to create object segments. Using the greyscale non‐skeletonized image Y, the image is denoised by suppressing small features and subsequently blurred using a Gaussian filter to smooth the pixel intensity in the stain region. This helps the object detection recognize individual cells and reduce over‐segmentation. The object detection algorithm uses the CellProfiler IdentifyPrimaryObjects module. This method does not require nuclear stain images or phase contrast cell images.

Further mitochondrial morphology analysis was performed using the MitoCellPhe analyzer. The morphology analysis can be computed on object morphological skeletons or a single aggregate morphological skeleton representing the stained sample. The morphology analysis can be run on batches of morphological skeletons by specifying the root directory. The output contains the file names along with each of the computed parameters detailed in Table S1. The morphology analysis output includes the skeleton area, the count of rods, count of punctates, punctate percentage, rod percentage, network percentage, punctate length, rod length, network length, mean rod length, total network branch count, mean network branch count, mean network branch, mean network length, all branch count, mean length of all branches, mean network, and rod length.

### 2.5 Statistical analysis

To ensure scientific rigor, a total of 50 cells across 3–4 biological replicates were used for the analysis related to human fibroblasts and hiPSCs. Since fibroblast cell lines have different cellular morphologies, the parameters generated were normalized by the cell surface area. Unlike the fibroblast cell lines, the hiPSC lines were not segmented. Normalization was applied for hiPSCs according to the number of nuclei in the analyzed areas generated by the software using MS Excel 2016. The data for the different parameters are presented as the mean ± standard deviation (SD) and were generated using MS Excel 2016. One‐way Anova or unpaired *t*‐tests were used accordingly to identify differences among specific groups. Post‐hoc Tukey HSD test was used to identify differences among specific groups. Data are presented as the mean ± standard deviation (SD) and were analyzed using the GraphPad Prism 8 program (GraphPad Software, San Diego, CA, USA). A *p*‐value <0.05 was considered significant.

## 3 RESULTS

### 3.1 MitoCellPhe reveals a fragmented mitochondrial phenotype in human fibroblast cell lines with pathogenic mtDNA mutation (*T8993G*, *T9185C*) impacting complex V

**FIGURE 1:**
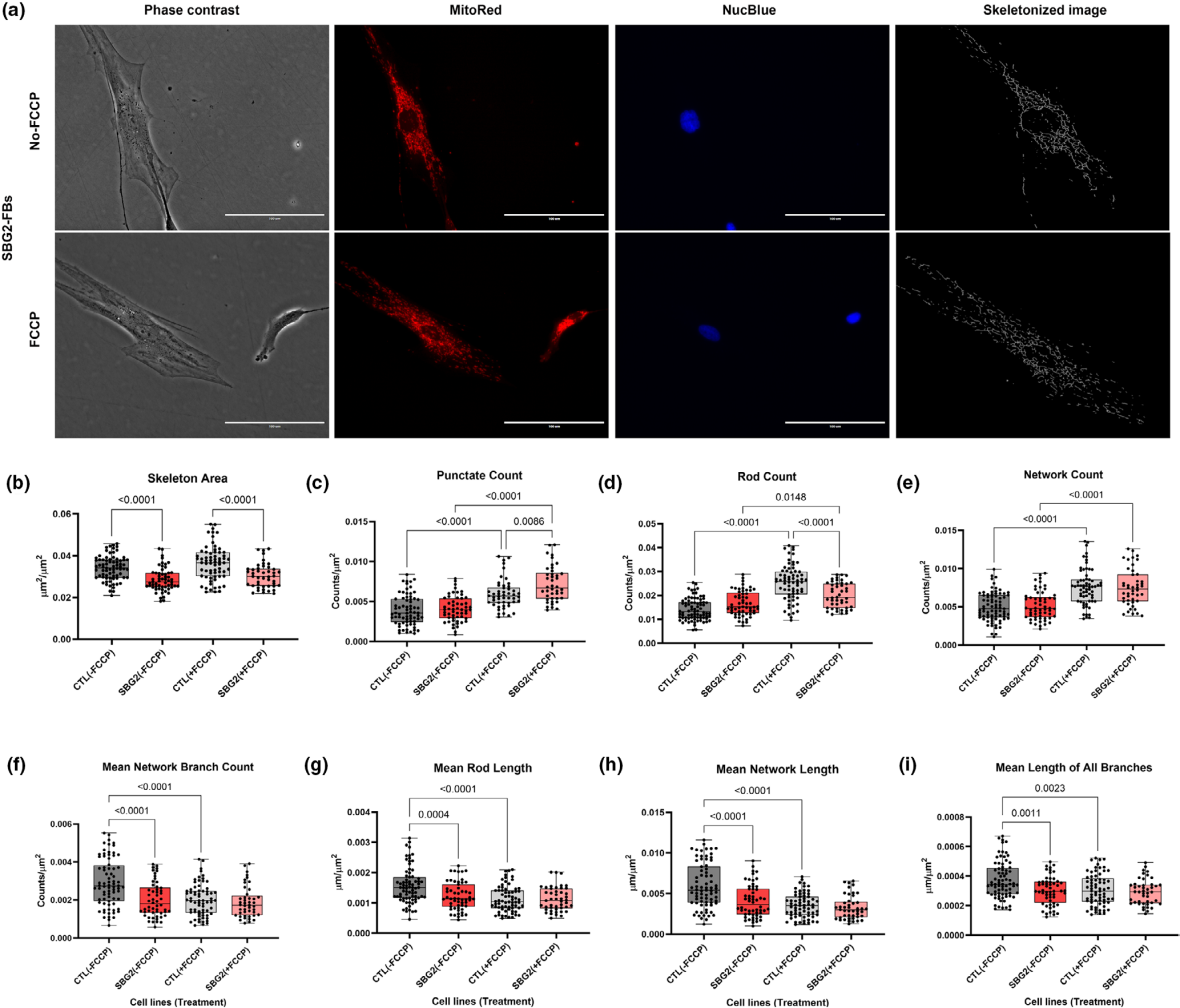
Mitochondrial morphology of diseased SBG2‐(*T8993G*) FB in the absence and presence of FCCP, in comparison to control (CTL) healthy BJ‐FBs. Representative Phase contrast, Mitotracker red, Nucblue, and Skeletonized images (a) are demonstrated. A Comparison in skeleton area (b), punctate count (c), rod count (d), network count (e), mean network branch count (f), mean rod length (g), mean network length (h), and mean lengths of all branches (i) relative to control under basal conditions, without and with FCCP treatment is illustrated. All data are representative of 10–14 images taken from three independent dishes/treatment groups. The bars represent minimum and maximum values, and each black dot represents a different data point. The dark and light gray bars represent the control fibroblast without and with FCCP treatment (FCCP vs. þ FCCP). The red and pink bars represent the SBG2‐FB (*T8993G*) without and with FCCP treatment (FCCP vs. þ FCCP). Scale bar = 100 μm.

**FIGURE 2:**
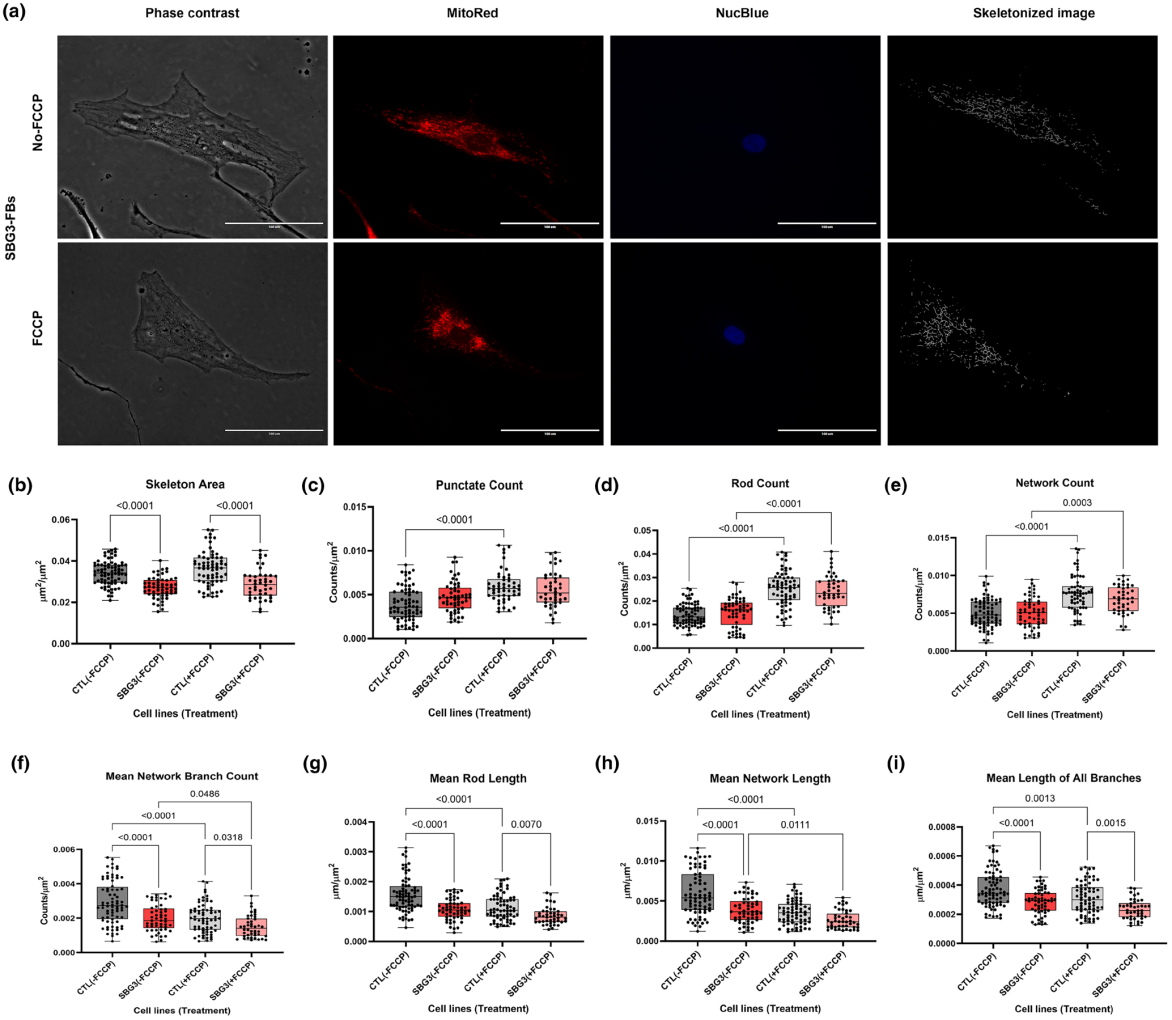
Mitochondrial morphology of diseased SBG3‐(*T9185C*) FB in the absence and presence of FCCP, in comparison to control (CTL) healthy BJ‐FBs. Representative Phase contrast, Mitotracker red, Nucblue, and Skeletonized images (a) are demonstrated. A Comparison in skeleton area (b), punctate count (c), rod count (d), network count (e), mean network branch count (f), mean rod length (g), mean network length (h), and mean lengths of all branches (i) relative to control under basal conditions, without and with FCCP treatment is illustrated. All data are representative of 10–14 images taken from three independent dishes/treatment groups. The bars represent minimum and maximum values, and each black dot represents a different data point. The dark and light gray bars represent the control fibroblast without and with FCCP treatment (FCCP vs. þ FCCP). The red and pink bars represent the SBG3‐FB (*T9185C*) without and with FCCP treatment (FCCP vs. þ FCCP). Scale bar = 100 μm.

Having confirmed that MitoCellPhe can generate detailed skeletons, which allow for quantification of the mitochondrial morphology in healthy differentiated and undifferentiated cell lines (Bakare, Meshrkey, et al., 2021), we proceeded to analyze differences between healthy control and two diseased fibroblast cell lines carrying mtDNA mutations impacting the subunit of the Complex V‐ATP synthase using Mitotracker red stain (Figures 1 and 2, Figure S9) and MitoCellPhe tool kit analysis (Bakare, Meshrkey, et al., 2021). We analyzed the healthy BJ control and two patient fibroblast cell lines (SBG2‐FB: *T8993G* and SBG3‐FB: *T9185C*) carrying some of the most prevalent pathogenic mtDNA mutations in patients with LS (Castagna et al., 2007; Debray et al., 2007). We analyzed mitochondrial morphology in the diseased SBG2‐(*T8993G*)‐FB and SBG3‐(*T9185C*)‐FB under basal conditions and after FCCP treatment.

The cell line SBG2‐(*T8993G*)‐FB demonstrated a significant decrease in skeleton area (*p* < 0.0001, by 17%) (Figure 1b), mean network branch count (*p* < 0.0001, by 31%) (Figure 1f), mean rod length (*p* = 0.0004, by 22%) (Figure 1g), mean network length (*p* < 0.0001, by 31%) (Figure 1h), and mean length of all branches (*p* = 0.0011, by 19%) (Figure 1i) relative to the BJ‐FB healthy control cell line. These results indicated that the cell line SBG2‐(*T8993G*)‐FB exhibited fragmented mitochondria compared to the healthy BJ‐FB control. The reduced skeleton area is indicative of fewer mitochondria in the SBG2‐(*T8993G*)‐FB cell line. The cell line SBG3‐(T9185C)‐FB demonstrated a significant decrease in skeleton area (*p* < 0.0001, by 21%) (Figure 2b), mean network branch count (*p* < 0.0001, by 31%) (Figure 2f), mean rod length (*p* < 0.0001, by 31%) (Figure 2g), mean network length (*p* < 0.0001, by 35%) (Figure 2h), mean length of all branches (*p* < 0.0001, by 24%) (Figure 2i) relative to the BJ‐FB healthy control cell line. The results were similar to those observed for SBG2‐(*T8993G*)‐FB, indicating fragmented mitochondria in this diseased cell line as well. Together, these results indicate that the cell lines with complex V defects exhibit fragmented mitochondria, reflecting a diseased state.

Mitochondrial morphology and dynamics are tightly linked to mitochondrial membrane potential (ΔΨ_m_) (Bakare, Daniel, et al., 2021; Bakare, Meshrkey, et al., 2021; Liesa & Shirihai, 2013). Therefore, we hypothesized that treating the cell lines with FCCP (trifluoromethoxy carbonyl cyanide phenylhydrazone), a mitochondrial uncoupler, would rapidly fragment the mitochondria, causing a concomitant increase in the number of punctates and rods in the diseased cell lines. Consistent with our hypothesis, we observed a significant increase in punctate count (*p* < 0.0001, by 76%) (Figure 1c), rod count (*p* = 0.0148, by 20%) (Figure 1d), in the SBG2‐(*T8993G*)‐FB FCCP‐treated cell line compared with an untreated SBG2‐(*T8993G*)‐FB cell line. We also observed a significant increase in rod counts (*p* < 0.0001, by 58%) (Figure 2d) in the SBG3‐(*T9185C*)‐FB FCCP‐treated cell line compared with an untreated SBG3‐(*T9185C*)‐FB cell line.

Our previous studies have shown that upon FCCP treatment, the SBG2‐(*T8993G*)‐FB cell line exhibits a decrease in maximal respiration, whereas the SBG3‐(*T9185C*)‐FB cell line exhibits an increase in maximal respiration, compared to the control BJ‐FB cell line, mimicking the physiological energy demand due to a defective ATP synthase (Bakare, Dean, et al., 2021). Therefore, we hypothesized that the diseased lines would exhibit differences in mitochondrial morphologies to reflect the increase in oxygen consumption upon treatment with FCCP. Our results demonstrate a significant increase in network count (*p* < 0.0001, by 46%) (Figure 1e) and a decrease in rod percentage (*p* < 0.0001, by 11%) (Figure S1D), in the SBG2‐(*T8993G*)‐FB FCCP‐treated cell line compared with the untreated SBG2‐(*T8993G*)‐FB cell line. We also observed a significant increase in network count (*p* = 0.0003, by 33%) (Figure 2e) and rod percentage (*p* = 0.0014, by 12%) (Figure S2D), in the SBG3‐(T*9185C*)‐FB FCCP‐treated cell line compared with an untreated SBG3‐(T9185C)‐FB cell line. In a previous study with another LS (SBG1‐(*T8993G*)‐FB) cell line carrying the same mutation as SBG2, we observed an increase in punctate count, rod count, and network count, with a decrease in mean network branch count and mean network length upon FCCP treatment. One possible explanation for the opposite results in the two cell lines concerning the rod percentages is the presence of an additional uncoupling defect in the SBG2‐(*T8993G*)‐FB cell line.

### 3.2 MitoCellPhe reveals dynamic mitochondrial network changes in human fibroblasts with pathogenic mtDNA mutation ( *T10158C* , *T12706C* ) impacting complex I

**FIGURE 3:**
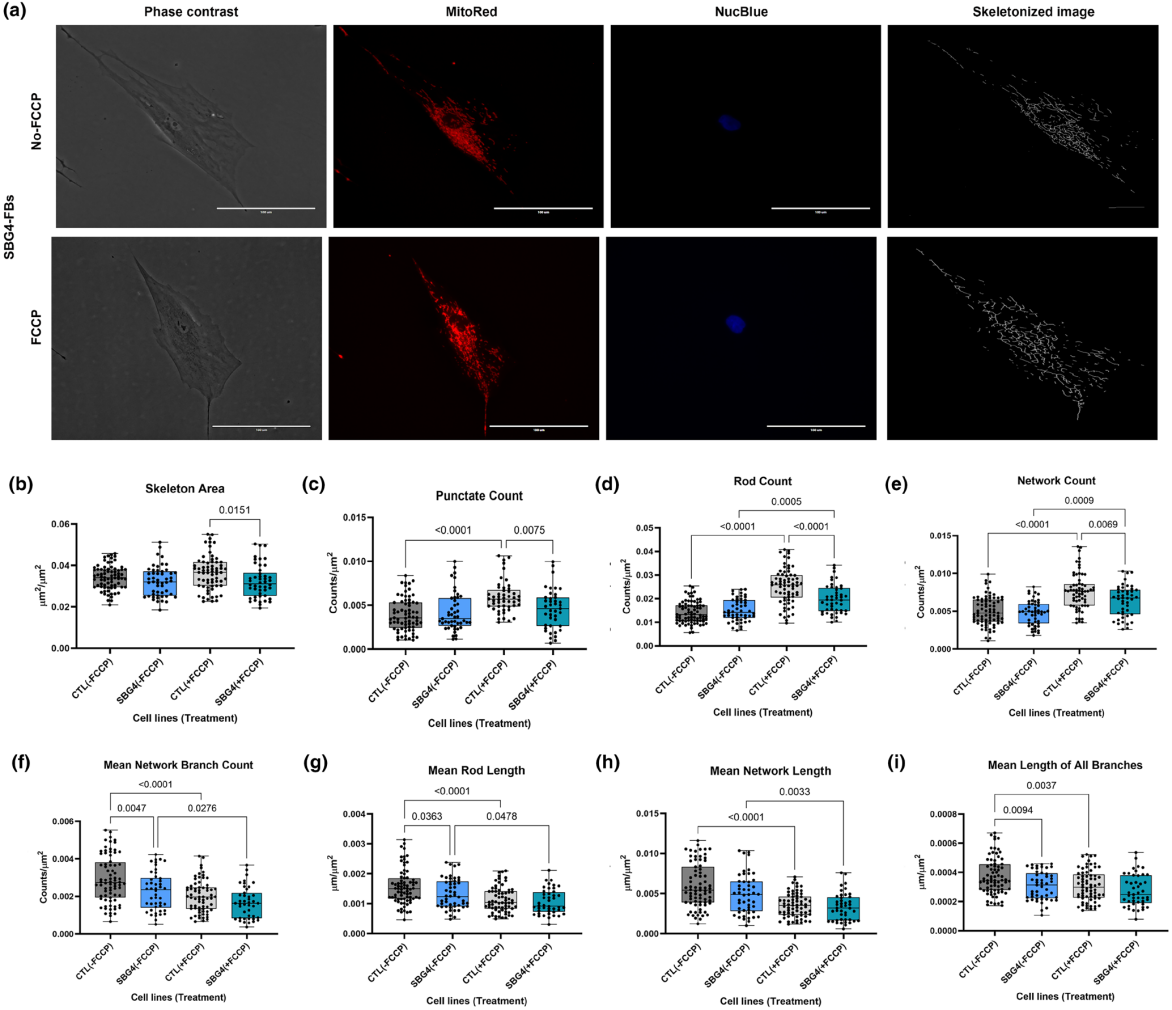
Mitochondrial morphology of diseased SBG4‐(*T10158C*) FB in the absence and presence of FCCP, in comparison to control (CTL) healthy BJ‐FBs. Representative Phase contrast, Mitotracker red, Nucblue, and Skeletonized images (a) are demonstrated. A Comparison in skeleton area (b), punctate count (c), rod count (d), network count (e), mean network branch count (f), mean rod length (g), mean network length (h), and mean lengths of all branches (i) relative to control under basal conditions, without and with FCCP treatment is illustrated. All data are representative of 10–14 images taken from three independent dishes/treatment groups. The bars represent minimum and maximum values, and each black dot represents a different data point. The dark and light gray bars represent the control fibroblast without and with FCCP treatment (FCCP vs. þ FCCP). The blue and green bars represent the SBG4‐FB (*T10158C*) without and with FCCP treatment (FCCP vs. þ FCCP). Scale bar = 100 μm.

**FIGURE 4:**
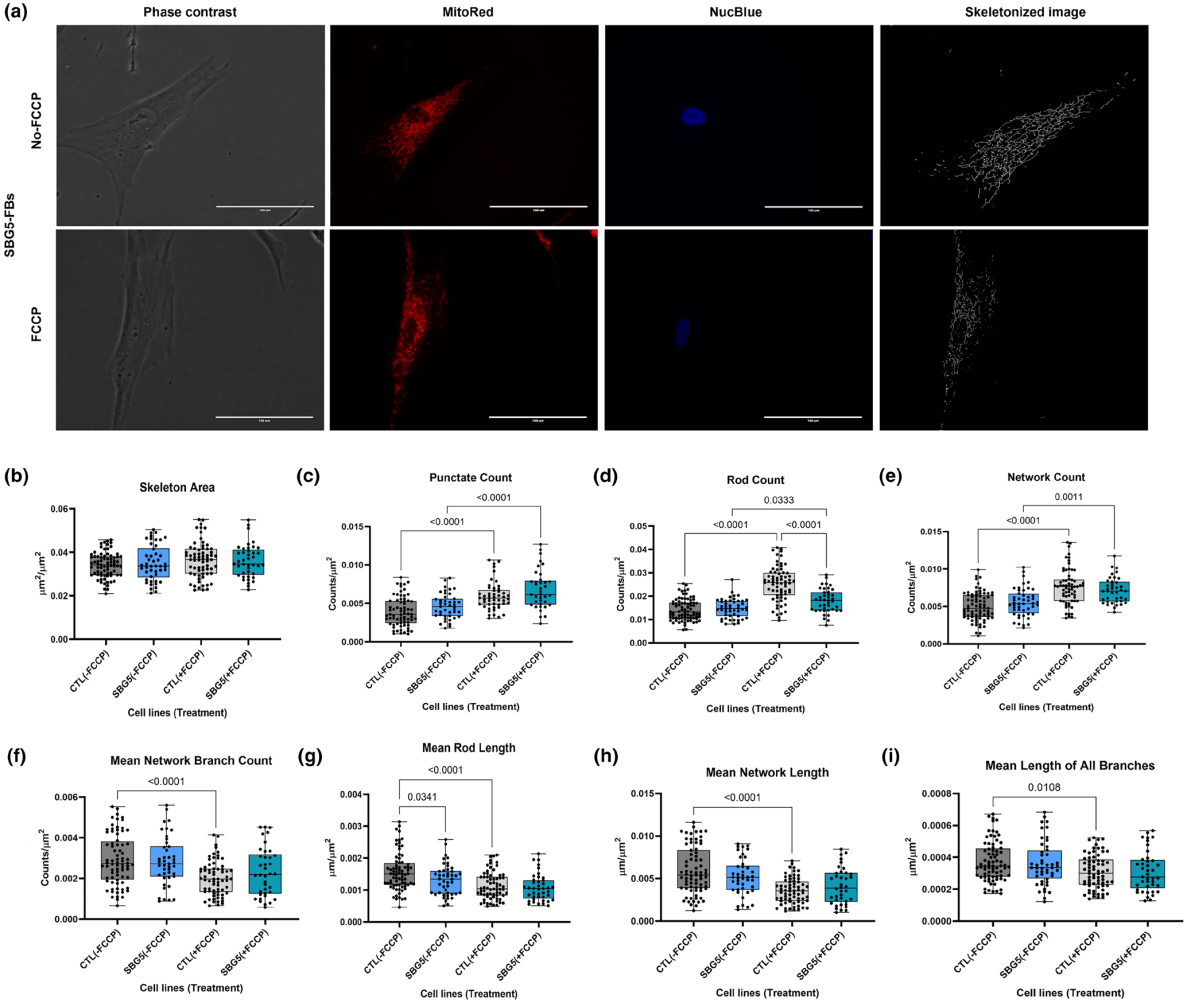
Mitochondrial morphology of diseased SBG5‐(*T12706C*) FB in the absence and presence of FCCP, in comparison to control (CTL) healthy BJ‐FBs. Representative Phase contrast, Mitotracker Red, Nucblue, and Skeletonized images (a) are demonstrated. A Comparison in skeleton area (b), punctate count (c), rod count (d), network count (e), mean network branch count (f), mean rod length (g), mean network length (h), and mean lengths of all branches (i) relative to control under basal conditions, without and with FCCP treatment is illustrated. All data are representative of 10–14 images taken from three independent dishes/treatment groups. The bars represent minimum and maximum values, and each black dot represents a different data point. The dark and light gray bars represent the control fibroblast without and with FCCP treatment (FCCP vs. þ FCCP). The blue and green bars represent the SBG5‐FB (*T12706C*) without and with FCCP treatment (FCCP vs. þ FCCP). Scale bar = 100 μm.

Next, we analyzed differences between healthy control and two diseased fibroblast cell lines carrying mtDNA mutation (SBG4‐FB: *T10158C*, and SBG5‐FB: *T12706C*), impacting the Complex I subunit using Mitotracker red stain (Figures 3 and 4, Figures S3 and S4). The cell line SBG4‐(T10158C)‐FB demonstrated a statistically significant decrease in mean network branch count (*p* = 0.0047, by 22%) (Figure 3f), mean rod length (*p* = 0.0363, by 16%) (Figure 3g), mean length of all branches (*p* = 0.0094, by 17%) (Figure 3i), mean network branch length (*p* = 0.0159, by 7%) (Figure S3F), mean network and rod length (*p* = 0.0029, by 28%) (Figure S3G), compared to the healthy control BJ‐FB cell line. The cell line SBG5‐(*T12706C*)‐FB demonstrated a statistically significant decrease in mean rod length (*p* = 0.0341, by 16%) (Figure 4g) and a statistically significant increase in total network branch count (*p* = 0.0457, by 14%) (Figure S4A), compared to the healthy control BJ‐FB cell line.

Next, both diseased cell lines were treated with FCCP to assess differential alterations in mitochondrial morphologies. We observed a statistically significant increase in rod count (*p* = 0.0005, by 31%) (Figure 3d), and network count (*p* = 0.0009, by 33%) (Figure 3e) in the SBG4‐(*T10158C*)‐FB FCCP‐treated cell line compared with an untreated SBG4‐(*T10158C*)‐FB cell line. We also observed a statistically significant decrease in mean network branch count (*p* = 0.0276, by 27%) (Figure 3f), mean rod length (p = 0.0478, by 20%) (Figure 3g), mean network length (p = 0.0033, by 7%) (Figure 3h), and mean network and rod length (p = 0.0166, by 28%) (Figure S3G) in the SBG4‐(T10158C)‐FB FCCP‐treated cell line compared with an untreated SBG4‐(*T10158C*)‐FB cell line. However, we observed a statistically significant increase in punctate count (*p* < 0.0001, by 47%) (Figure 4c), rod count (*p* = 0.033, by 22%) (Figure 4d), and network count (*p* = 0.0011, by 29%) (Figure 4e) in the SBG5‐(*T12706C*)‐FB FCCP‐treated cell line compared with an untreated SBG5‐(*T12706C*)‐FB cell line.

Overall, our results indicate that there was a decrease in the average number of branches per network, accompanied by a shortening of the branches within the networks and in individual rods in the SBG4‐(*T10158C*)‐FB cell line compared to the control BJ‐FB cell line. This change in morphology was further exacerbated upon FCCP treatment, reflected by an increase in the number of rods and networks. However, the change in mitochondrial morphology in SBG5‐(*T12706C*)‐FB cell lines was only observed upon FCCP treatment, which showed rapid fragmentation of mitochondria based on an increase in the number of punctate, rod, and network.

### 3.3 Variable morphologies that are variant‐specific in hiPSCs impacting complex V with *T8993G* , *T9185C* mutations

**FIGURE 5:**
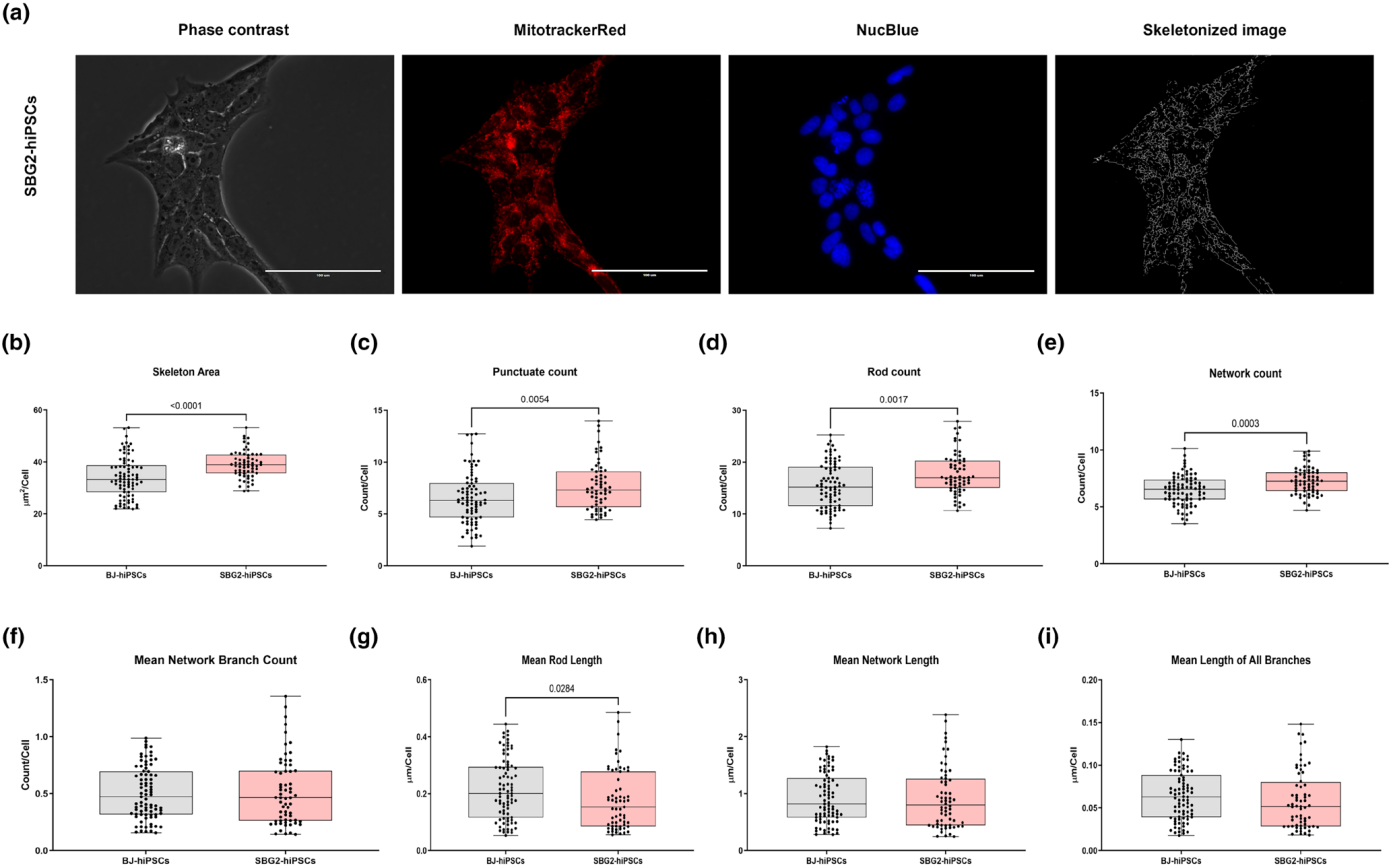
Mitochondrial morphology of SBG2‐(*T8993G*) hiPSC, in comparison to healthy control BJ‐hiPSC. Representative Phase contrast, Mitotracker Red, Nucblue, and Skeletonized images (a) are demonstrated. Different mitochondrial morphological parameters were determined and analyzed in comparison with the BJ‐hiPSC (control cell line) using MitoCellPhe to quantify the mitochondrial skeleton area (b), punctate count (c), rod count (d), network count (e), mean network branch count (f), mean rod length (g), mean network length (h), and mean lengths of all branches (i). All data are representative of five to seven analyzed images obtained from seven to nine independent dishes from three independent experiments. The bars represent minimum and maximum values, including all points, and each black dot represents a different data point. The gray bars represent the BJ‐control hiPSC, whereas the pink bars represent SBG2‐(*T8993G*)‐hiPSC. Scale bar = 100 μm.

**FIGURE 6:**
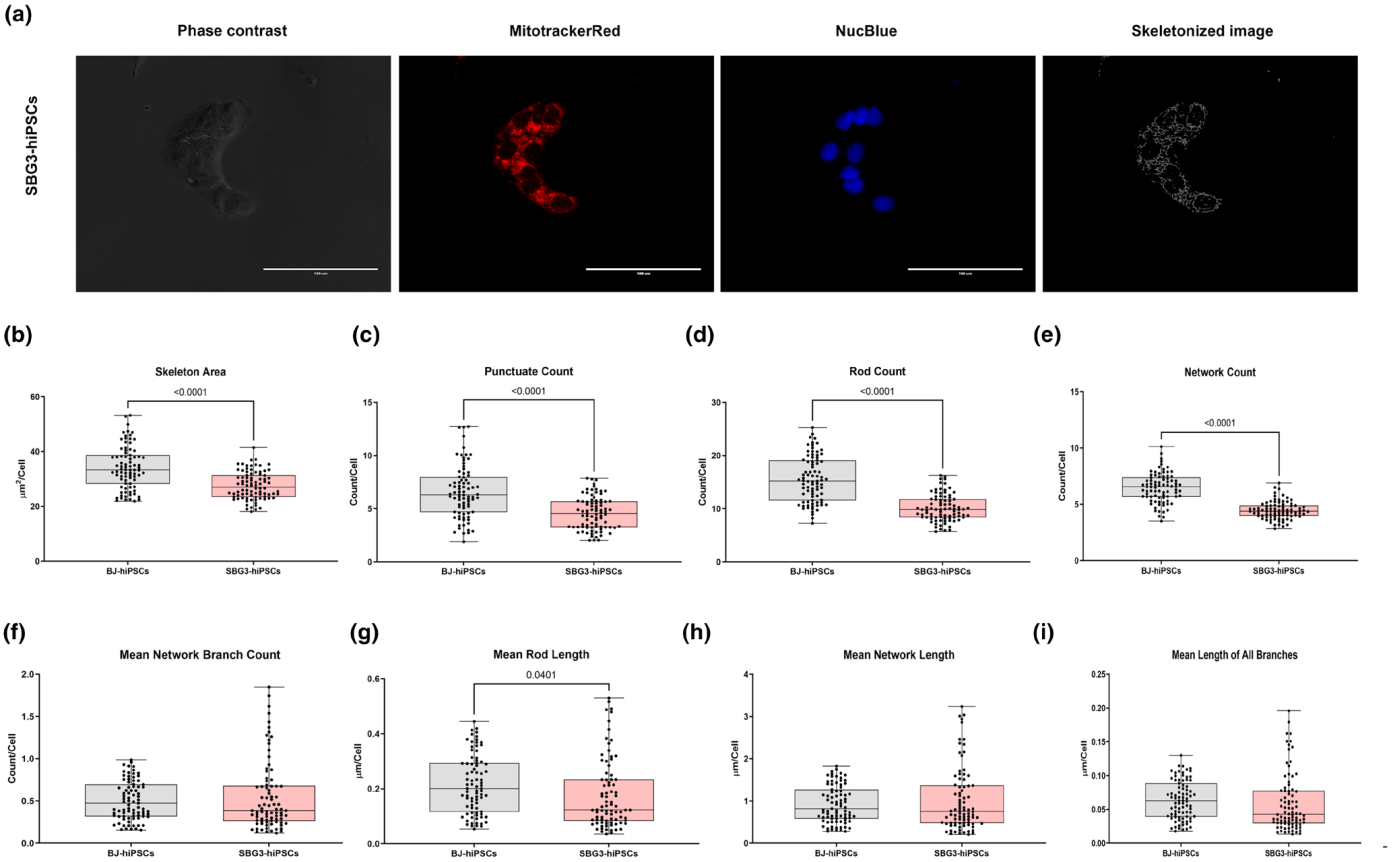
Mitochondrial morphology of SBG3‐(*T9185C*)‐hiPSC, in comparison to healthy control BJ‐hiPSC. Representative Phase contrast, Mitotracker Red, Nucblue, and Skeletonized images (a) are demonstrated. Different mitochondrial morphological parameters were determined and analyzed in comparison with the BJ‐hiPSC (control cell line) using MitoCellPhe to quantify the mitochondrial skeleton area (b), punctate count (c), rod count (d), network count (e), mean network branch count (f), mean rod length (g), mean network length (h), and mean lengths of all branches (i). All data are representative of five to seven analyzed images obtained from seven to nine independent dishes from three independent experiments. The bars represent minimum and maximum values, including all points, and each black dot represents a different data point. The gray bars represent the BJ‐control hiPSC, whereas the pink bars represent the SBG3‐(*T9185C*)‐hiPSC. Scale bar = 100 μm.

In a previous study, we determined the utility of the MitoCellPhe toolset to detect different mitochondrial morphologies in healthy control BJ‐hiPSCs and in complex V‐ATP synthase SBG1‐hiPSCs harboring the *T8993G* mutation (Bakare, Meshrkey, et al., 2021). We hypothesized that branching reflected the balance between fusion (which increases connectivity & the number of branched points, density) and fission (which reduces connectivity and increases the number of fragmented outcomes). Therefore, high branch counts indicated greater mitochondrial fusion, higher network complexity, and often increased oxidative or metabolic activity. Similarly, low branch counts indicated a fissioning state of the mitochondria, and higher “punctate” counts often suggested an increase in fragmented mitochondria accompanied by increased cellular stress or apoptosis signaling activity. Two patient‐derived hiPSCs harboring the most mutations prevalent in patients with LS (SBG2: *T8993G*; SBG3: *T9185C*) (Castagna et al., 2007) were stained with Mitotracker red, skeletonized (Figures 5a and 6a), and analyzed using MitoCellPhe. The cell line SBG2‐(*T8993G*)‐hiPSC exhibited a statistically significant increase in skeletal area (*p* < 0.0001, by 14%) (Figure 5b), punctate counts (*p* = 0.0054, by 19%) (Figure 5c), rod counts (*p* = 0.0017, by 16%) (Figure 5d), network count (*p* = 0.0003, by 12%) (Figure 5e), total network branch count (*p* < 0.0001, by 20%) (Figure S5A), all branch count (*p* < 0.0001, by 19%) (Figure S5B) compared to the control BJ‐hiPSC cell line. We also observed a statistically significant decrease in mean rod length (*p* = 0.0284, by 16%) (Figure 5g), compared to the control BJ‐hiPSC cell line. The cell line SBG3‐(*T9185C*)‐hiPSC exhibited a statistically significant decrease in the skeletal area (*p* < 0.0001, by 19%) (Figure 6b), punctate count (*p* < 0.0001, by 29%) (Figure 6C), rod count (*p* < 0.0001, by 35%) (Figure 6d), network count (*p* < 0.0001, by 31%) (Figure 6e), mean rod length (*p* = 0.0401, by 12%) (Figure 6G), total network branch count (*p* < 0.0001, by 18%) (Figure S6A) and all branch count (*p* < 0.0001, by 20%) (Figure S6B), compared to the control BJ‐hiPSC cell line. These results support our hypothesis that increased connectivity and the number of branches and related parameters indicate a more fused morphology in the SBG2‐(*T8993G*)‐hiPSC cell line. In contrast, a significant reduction in branched connectivity and related parameters indicated a more fragmented morphology in the SBG3‐(*T9185C*)‐hiPSC cell line. It also implies that the specific mtDNA mutations *T8993G* and *T9185C* impacted the function of Complex V subunit differently.

### 3.4 hiPSCs with *T10158C* , *T12706C* mutations impacting complex I exhibit higher branch length measurements with other variable mitochondrial phenotype characteristics

**FIGURE 7:**
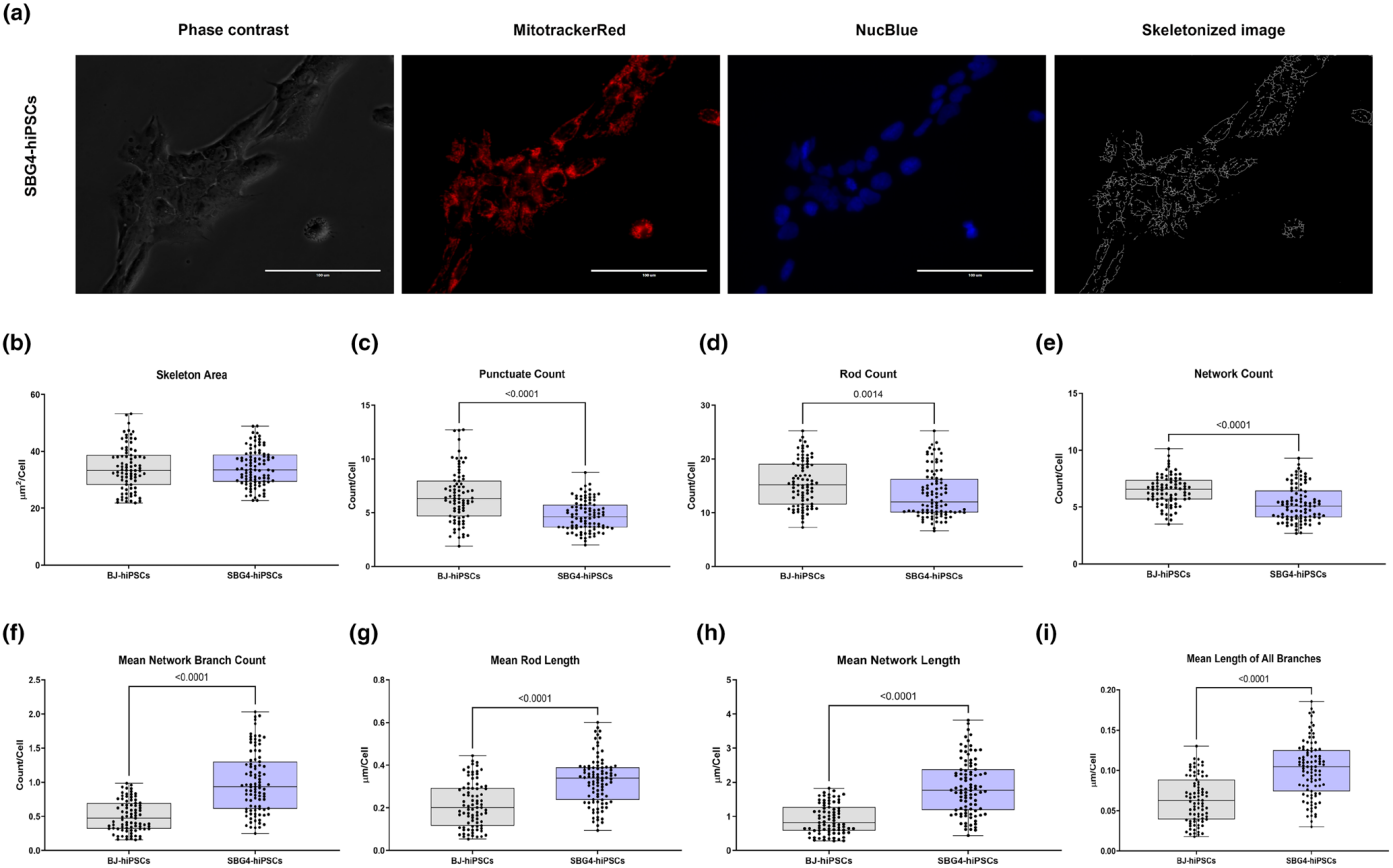
Mitochondrial morphology of SBG4‐(*T10158C*)‐hiPSC, in comparison to healthy control BJ‐hiPSC. Representative Phase contrast, Mitotracker Red, Nucblue, and Skeletonized images (a) are demonstrated. Different mitochondrial morphological parameters were determined and analyzed in comparison with the BJ‐hiPSC (control cell line) using MitoCellPhe to quantify the mitochondrial skeleton area (b), punctate count (c), rod count (d), network count (e), mean network branch count (f), mean rod length (g), mean network length (h), and mean lengths of all branches (i). All data are representative of five to seven analyzed images obtained from seven to nine independent dishes from three independent experiments. The bars represent minimum and maximum values, including all points, and each black dot represents a different data point. The gray bars represent the BJ‐control hiPSC, whereas the blue bars represent the SBG4‐(*T10158C*)‐hiPSC. Scale bar = 100 μm.

**FIGURE 8:**
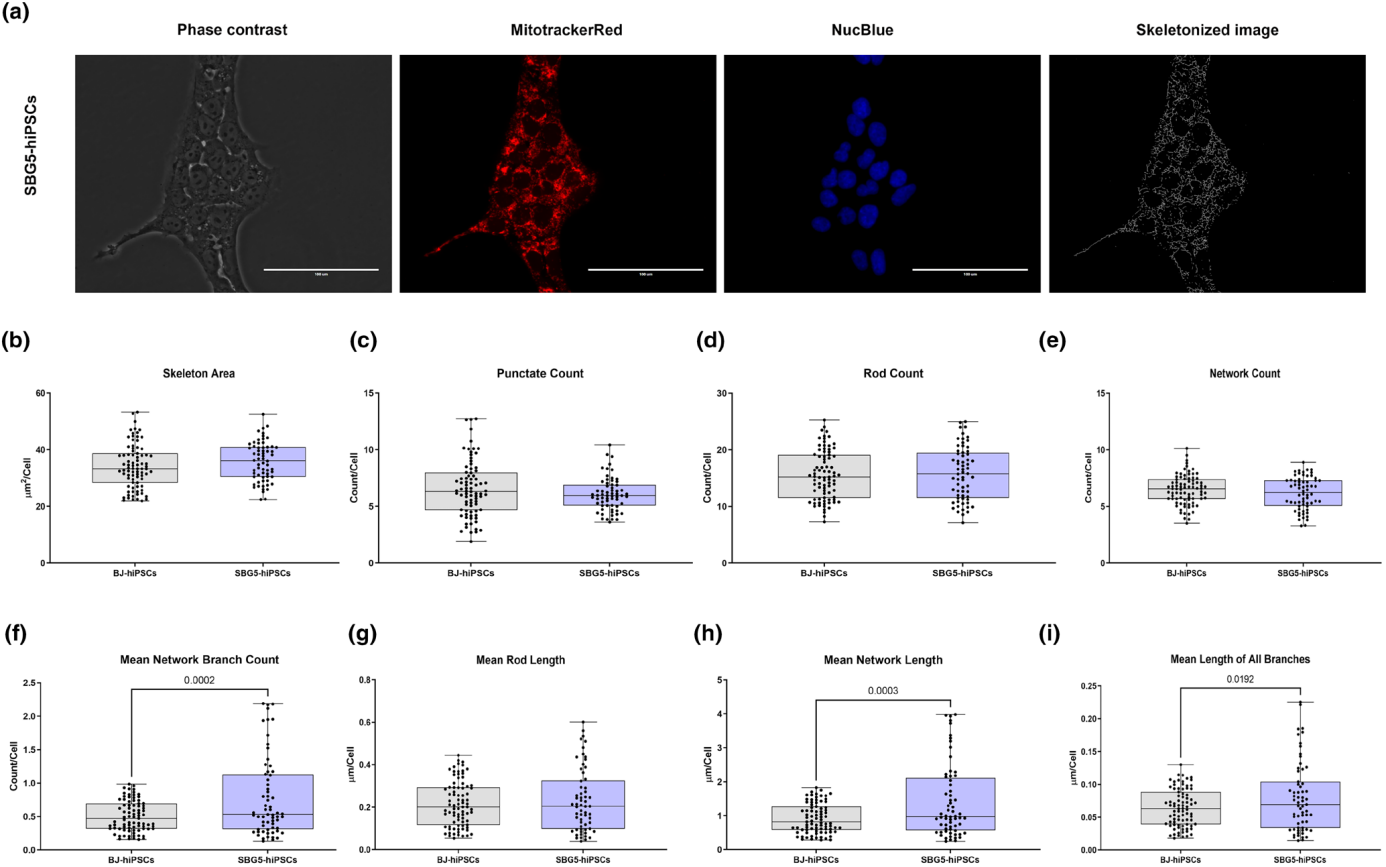
Mitochondrial morphology of SBG5‐(*T12706C*)‐hiPSC, in comparison to healthy control BJ‐hiPSC. Representative Phase contrast, Mitotracker Red, Nucblue, and Skeletonized images (a) are demonstrated. Different mitochondrial morphological parameters were determined and analyzed in comparison with the BJ‐hiPSC (control cell line) using MitoCellPhe to quantify the mitochondrial skeleton area (b), punctate count (c), rod count (d), network count (e), mean network branch count (f), mean rod length (g), mean network length (h), and mean lengths of all branches (i). All data are representative of five to seven analyzed images obtained from seven to nine independent dishes from three independent experiments. The bars represent minimum and maximum values including all points, and each black dot represents a different data point. The gray bars represent the BJ‐control hiPSC, whereas the blue bars represent the SBG5‐(*T12706C*)‐hiPSC. Scale bar = 100 μm.

To determine the specificity of mutations in other complexes that may affect mitochondrial morphology and, ultimately, mitochondrial function in patient‐derived LS‐hiPSCs, we evaluated mitochondrial morphologies in two diseased hiPSC lines harboring a complex I mutation in the electron transport chain. The SBG4‐hiPSC line has been reprogrammed from a patient fibroblast cell line with a *T10158C* point mutation in the MTND3 gene, and SBG5‐hiPSC has been derived from a patient fibroblast cell line with the *T12706C* point mutation in the MTND5 gene. Studies have demonstrated the presence of these mutations in patients with LS or LS‐like syndromes (Bakare, Dean, et al., 2021; Mitchell, 1966; Zhadanov et al., 2007). We observed a statistically significant decrease in punctate count (*p* < 0.0001; by 28.9%) (Figure 7c), rod count (*p* = 0.0014; by 12.64%) (Figure 7d), and network count (*p* < 0.0001; by 18.27%) (Figure 7e) and punctate percentage (*p* = 0.0016; by 11%) (Figure S7C) in SBG4‐(*T10158C*)‐hiPSC when compared with the healthy control BJ‐hiPSC. We also identified a significant increase in the mean network branch count (*p* < 0.0001; by 97%) (Figure 7f); mean rod length (*p* < 0.0001; by 50.62%) (Figure 7g), mean network length (*p* < 0.0001; by 102.69%) (Figure 7h), mean length of all branches (*p* < 0.0001; by 58.55%) (Figure 7i), rod percentage (*p* = 0.0002; by 9.4%) (Figure S7D); the mean network branch length (p < 0.0001; by 58.05%) (Figure S7F), and the mean network and rod length (*p* < 0.0001; by 91.31%) (Figure S7G) in SBG4‐(T10158C)‐hiPSC when compared with the healthy control BJ‐hiPSC. The cell line SBG5‐(T12706C)‐hiPSCs exhibited a statistically significant increase in the mean length of all branches (*p* = 0.0192; by 29.53%) (Figure 8i), mean network length (*p* = 0.0003; by 53.03%) (Figure 8h), mean network branch count (*p* = 0.0002; by 30.68%) (Figure 8f), the mean network branch length (*p* = 0.0072; by 20%) (Figure S8F), the mean network and rod length (*p* = 0.0015; by 49.45%) (Figure S8G), in SBG5‐(T12706C)‐hiPSCs when compared with healthy control BJ‐hiPSCs. We also observed a significant decrease in network percentage (*p* = 0.0423; by 3.74%) (Figure S8E) in SBG5‐(*T12706C*)‐hiPSCs when compared with healthy control BJ‐hiPSCs. Overall, the increased length measurements in both SBG4‐(*T10158C*) and SBG5‐(*T12706C*)‐hiPSCs suggest the presence of fused mitochondria. However, the extent of the other phenotype associated with other observations, such as rod, punctate, and network count, may be dependent either on the mutation site or specific cellular response according to the metabolic demands.

## 4 DISCUSSION

Mitochondria are dynamic organelles that perform a plethora of cellular processes, which depend on the tight regulation of their shapes and ultrastructure (JG et al., 2013; Sprenger & Langer, 2019). Understanding the dynamic nature of mitochondria in healthy and diseased states can be beneficial in finding targeted therapies for disorders that result from mitochondrial dysfunction. We have previously demonstrated that MitoCellPhe is a useful tool for quantifying mitochondrial dynamics in single or clustered cells (Bakare, Meshrkey, et al., 2021). Further, we have shown in a previous study the dynamic nature of the mitochondria in patient cell lines carrying mutations involved in various mitochondrial disorders (Bakare, Daniel, et al., 2021; Bakare, Meshrkey, et al., 2021; Meshrkey et al., 2021). In this study, we explore further the ability of MitoCellPhe to quantify mitochondrial dynamics in patient fibroblast (differentiated) and hiPSCs (undifferentiated) in the context of disease (Tables 1 and 2).

**TABLE 1:** Summary of mitochondrial morphology observed in diseased fibroblasts.

Mitochondrial morphology	Fibroblast (differentiated/individual cells)			
	SBG2‐FB	SBG3‐FB	SBG4‐FB	SBG5‐FB
Skeletal area	↓ (<0.0001)	↓ (<0.0001)	↓	↑
Punctuate count	↑	↑	↑	↑
Rod count	↑	↑	↑	↑
Network count	↑	↑	↓	↑
Mean network branch count	↓ (<0.0001)	↓ (<0.0001)	↓ (0.0047)	↓
Mean rod length	↓ (0.0004)	↓ (<0.0001)	↓ (0.0363)	↓ (0.0341)
Mean Network length	↓ (<0.0001)	↓ (<0.0001)	↓	↓
Mean length of all branches	↓ (0.0011)	↓ (<0.0001)	↓ (0.0094)	↓
Total network branch count	↓ (<0.0001)	↓ (0.0006)	↓	↑ (0.0457)
All branch count	↓ (0.0011)	↓ (0.0029)	↓	↑
Punctuate percentage	↓	↑	↓	↑
Rod percentage	↑ (0.0269)	↓	↑	↓
Network percentage	↓	↓	↓	↑
Mean network branch length	↓ (0.005)	↓ (0.0003)	↓ (0.0159)	↓
Mean network and rod length	↓ (<0.0001)	↓ (<0.0001)	↓ (0.0029)	↓

*Note*: Comparison of mitochondrial morphology parameters obtained from MitoCellPhe of the diseased fibroblast cells in comparison to the control cell line (BJ‐FB) under basal conditions. ↑↓ means increase or decrease in value. *p*‐values are noted in parenthesis.

**TABLE 2:** Summary of mitochondrial morphology observed in diseased iPSCs.

Mitochondrial morphology	Induced pluripotent stem cells (undifferentiated/ cluster cells)			
	SBG2‐hiPSC	SBG3‐hiPSC	SBG4‐hiPSC	SBG5‐hiPSC
Skeletal area	↑ (<0.0001)	↓ (<0.0001)	↑	↑
Punctuate count	↑ (0.0054)	↓ (<0.0001)	↓ (<0.0001)	↓
Rod count	↑ (0.0017)	↓ (<0.0001)	↓ (0.0014)	↑
Network count	↑ (0.0003)	↓ (<0.0001)	↓ (<0.0001)	↓
Mean network branch count	↑	↑	↑ (<0.0001)	↑ (0.0002)
Mean rod length	↓ (0.0284)	↓ (0.0401)	↑ (<0.0001)	↑
Mean Network length	↓	↑	↑ (<0.0001)	↑ (0.0003)
Mean length of all branches	↓	↓	↑ (<0.0001)	↑ (0.0192)
Total network branch count	↑ (<0.0001)	↓ (<0.0001)	↓	↑
All branch count	↑ (<0.0001)	↓ (<0.0001)	↓	↑
Punctuate percentage	↑	↑	↓ (0.0016)	↓
Rod percentage	↑	↓	↑ (0.0002)	↑
Network percentage	↓	↑	↓	↓ (0.0423)
Mean network branch length	↓	↓	↑ (<0.0001)	↑ (0.0072)
Mean network and rod length	↓	↑	↑ (<0.0001)	↑ (0.0015)

*Note*: Comparison of mitochondrial morphology parameters obtained from MitoCellPhe of the diseased undifferentiated hiPSCs in comparison to the control BJ‐hiPSC cell line under basal conditions. ↑↓ means an increase or decrease in value. *p*‐values are noted in parentheses.

First, we compared mitochondrial morphology between two fibroblast cell lines carrying mutations (*T8993G*, *T9185C*) impacting complex V of the mitochondria. We show that although these cell lines carry different point mutations, they exhibit a very similar mitochondrial morphology. Compared to the control cell line, both SBG2‐(*T8993G*)‐FB and SBG3‐(*T9185C*)‐FB have a fragmented morphology. These results are consistent with our previous study, where we showed that mutations in Complex V resulted in smaller, fragmented mitochondria (Bakare, Daniel, et al., 2021). When we treated these cell lines with FCCP to record their response to stress, both SBG2‐(*T8993G*)‐FB and SBG3‐(*T9185C*)‐FB responded accordingly with increases in network and rod counts. Although we reported previously that SBG2‐(*T8993G*)‐FB and SBG3‐(*T9185C*)‐FB had different compensatory responses because of the differences in a point mutation, we demonstrate in this study that MitoCellPhe is sensitive enough to pick up changes that were not picked up in the previous study (Bakare, Daniel, et al., 2021). Furthermore, we show that the difference in compensatory responses is associated more with homeostasis in the proportion of each mitochondrial structure rather than the absolute count. While the punctate% and network% significantly decreased in SBG3‐(*T9185C*)‐FB after FCCP treatment, it was the opposite for SBG2‐(*T8993G*)‐FB. Total network branch count was also significantly increased in SBG2‐(*T8993G*)‐FB, while it stayed the same in SBG3‐(*T9185C*)‐FB after FCCP treatment. These results further support the sensitivity of MitoCellPhe and support previous studies that have suggested that fragmentation is advantageous for uncoupled respiration (Liesa & Shirihai, 2013).

In the cell lines carrying mtDNA mutations (SBG4‐FB: *T10158C*, and SBG5‐FB: *T12706C*) impacting complex I, we observed that fragmentation was also the predominant change in the SBG4‐(*T10158C*)‐FB relative to the healthy control BJ‐FB cell line. However, in the SBG4‐FB cell line, fragmentation of networked mitochondria is prevalent, which is supported by the significant decrease in mean network branch count, mean length of all branches, mean network & rod length, and mean network branch length. Although there was no statistically significant change in several parameters in the SBG5‐(*T12706C*)‐FB cell line when compared with the control BJ‐FB cell line, the increase in the total network branch count suggests that this cell line exhibits a hyperfused morphology. This result is consistent with our previous report (Bakare, Daniel, et al., 2021) on the presence of hyperfused morphology with this specific cell line. FCCP treatment resulted in an overall increase in punctate, network, and rod counts for SBG5‐(*T12706C*)‐FB. Moreover, in the SBG4‐(*T10158C*)‐FB only network and rod count increased after FCCP treatment, while punctate counts remained the same as in the untreated group. We also observed a decrease in mean rod length, mean network length, and mean network & rod length in SBG4‐(*T10158C*)‐FB, as expected for cell lines undergoing fragmentation in response to FCCP treatment.

In addition, our findings are in line with previous findings where fragmented mitochondria are the predominant morphology in the diseased state (Liu & Hajnoczky, 2011; Tokuyama et al., 2020; Wu et al., 2011). Furthermore, the results suggest that it is not the number of fragmented mitochondria per se that is detrimental, but the proportion of mitochondria of a certain size that contributes to disease. This is supported by new findings in the literature that suggest that some types of fission result in mitophagy, while others lead to proliferation (Kleele et al., 2021). Future studies exploring this relationship could provide more pertinent information on the correlation between changes in mitochondrial morphologies and mitochondrial function as it relates to disease pathophysiology. Nevertheless, we have shown that MitoCellPhe can be used to explore differences in mitochondrial morphology in different diseased cell lines. Furthermore, we show that the sensitive nature and comprehensive data obtained from MitoCellPhe enable better comprehension of the relationship between mitochondrial dynamics and disease in differentiated cells such as fibroblasts.

Using the Mitocellphe tool, we were able to quantify in detail the different morphologies in undifferentiated cells of four reprogrammed LS: SBG2‐(*T8993G*), SBG3‐(*T9185C*), SBG4‐(*T10158C*), and SBG5‐(*T12706C*)‐hiPSCs. Across all four hiPSCs with different mtDNA variants, we observed and summarized the differences in the mitochondrial morphologies when compared with the healthy control BJ‐hiPSC. In computing the total area occupied by mitochondria, we observed an increase in SBG2‐(*T8993G*)‐hiPSC, with a decrease in total mitochondria in SBG3‐(*T9185C*)‐hiPSC. In SBG2‐(*T8993G*)‐hiPSC, which has a mutation in the ATP synthase complex ATP6 subunit, we noticed that cells tend to have more mitochondria and the tendency of these mitochondria to adapt to the fragmented phenotype. The latter observation was manifested by the significant increase in the count of punctate, rods, and networks. However, the mitochondria in SBG2‐(*T8993G*)‐hiPSC preferred to maintain a smaller rod morphology rather than the network or the punctate form, manifested by the decrease in the mean rod length, while punctate and network percentage were comparable to the control BJ‐hiPSC line. This specific identification is a practical usage for the MitoCellPhe tool, as we can distinguish the punctate and the rods as separate entities (Bakare, Meshrkey, et al., 2021). In the SBG3‐(*T9185C*)‐hiPSCs, we noticed a decrease in most of the parameters as evaluated by the MitoCellPhe tool, which could be derived initially from the lower mitochondria occupying the skeletal area. Generally, we noted that the difference between SBG2‐(*T8993G*)‐hiPSC, SBG3‐(*T9185C*)‐hiPSC, and the control BJ‐hiPSC is minimal and may be related to the initial area occupied by mitochondria. In addition, although both cell lines have the same subunit mutation in complex V‐ATP synthase, they show a different pattern for mitochondrial morphology, denoting that other factors may play an important role in changing the mitochondrial morphology according to the metabolic needs of the cells. These factors could include the specific point mutation site, the mutation load, and the environmental factors.

In SBG4‐(*T10158C*)‐hiPSC, and in SBG5‐(T12706C)‐hiPSC, we observed a potential for hyperfusion in both cell lines, represented by the increase in the length of measures such as the mean rod length, mean network length, mean branch length, mean network branch length, and mean network branch count. In SBG4‐(*T10158C*)‐hiPSC, the increase in length measurements was associated with a decrease in the number of punctates, rods, and networks, with an increase in the percentage of rods, indicating also the hyperfused state adopted by the mitochondria in the cells, which may be a sign of abnormal mitochondrial phenotypes (Philley et al., 2016). Fragmented mitochondria usually exist in a severe mitochondrial disease status (Knott et al., 2008; Wu et al., 2011; Zemirli et al., 2018) However, hyperfusion with an increase in mitochondrial network size is also commonly observed in complex I mutation, particularly with less severe conditions (Koopman, Visch, et al., 2005). Indeed, long‐term application of the complex I inhibitor, rotenone, in healthy fibroblasts increases mitochondrial branching (Koopman, Verkaart, et al., 2005). Cells with mitochondrial mutation may adapt this morphology in mild stress as a buffering mechanism, as generally, elongated mitochondria are resistant to apoptosis and mitophagy (Vigie & Camougrand, 2017; Zemirli et al., 2018).

Overall, the comprehensive output provided by MitoCellPhe helped in detecting the subtle differences that exist in the pluripotent state of our reprogrammed diseased cell lines, and more notably, identify the preferences of cells for rods, punctate, or network morphology. This study characterized mitochondrial morphology in hiPSCs and parental fibroblasts across four patients exhibiting LS or LS‐like syndromes. The results illustrate the existence of subtle differences in mitochondrial morphologies in different LS‐hiPSCs with different mtDNA mutation variants. From these acquired results, we can indicate the presence of variation between the different cell lines that may be variant‐specific but also due to other factors. One of these factors is a direct correlation between mtDNA disease severity and clinical outcomes, to the degree of heteroplasmy and the mutation load. Our preliminary data based on comprehensive next‐generation sequencing analysis indicate varying heteroplasmy levels (between 0.4% and 96%) in the different LS‐hiPSCs. Although the low levels of mutation burden existed in two of the hiPSCs SBG4‐(T10158C)‐hiPSC‐0.5% and SBG5‐(*T12706C*)‐hiPSC‐0.8%, the MitoCellPhe toolkit was sensitive enough to detect mitochondria with elongated branches, indicating a tendency towards a hyperfused state. These results prove the involvement of other factors such as environmental factors and epigenetics that may require further investigations and could play an important role in disease pathway and severity. When the cell lines are treated with FCCP, we noted that some of the diseased cell lines exhibited the same response to FCCP as the healthy control BJ‐FB lines. We have observed similar trends with basal (oxygen consumption rate) and maximal respiration values using a Seahorse Flux Analyzer, thus indicating that variable respiration rates are dependent on specific mtDNA mutations in the different disease fibroblasts (Bakare, Daniel, et al., 2021). To better comprehend the clinical presentation associated with the pathogenic mtDNA mutations and the biochemical defects in mitochondrial disease, we have proposed a composite bioenergetic health index ratio (BHI) as an approach to assessing mitochondrial health (Bakare, Dean, et al., 2021). Our results indicate that during stress triggered by specific pathogenic mtDNA variants or other factors, cells SBG4‐FB (*T10158C*) with high spare reserve capacity (SRC), low heteroplasmy, and high composite BHI ratio exhibit delayed onset and mild clinical symptoms. However, as the SRC and composite BHI ratio decrease, cells SBG5‐FB (*T12706C*) are unable to handle stress and exhibit early‐onset and severe clinical symptoms despite low heteroplasmy levels. Whereas cells carrying pathogenic disease mtDNA variants in ATP6 gene SBG2‐FB (T8993G) and SBG3‐FB (*T9185C*) exhibit very high heteroplasmy levels and lower composite BHI ratio when compared with control BJ‐FB and can be grouped as “intermediate” in disease severity. In general, diseased cell lines under basal conditions tend to have fewer respiring mitochondria with small branches and fragmented networks, while hyperfusion could serve as a compensatory mechanism. Finally, mitochondrial dynamics are involved in the pathogenesis of many diseases, such as neurodegenerative, cardiovascular, cancer, and metabolic disorders (Duchen, 2004; Wallace, 2012). Thus, enabling researchers from these fields to investigate mitochondrial dynamics depends upon the accessibility of a feasible system to quantify and describe mitochondrial phenotype. Hence, we have shown that MitoCellPhe can be used to explore differences in mitochondrial morphology in different diseased cell lines. Furthermore, we show that the sensitive nature and comprehensive data obtained from MitoCellPhe enable a better conception of the relationship between mitochondrial dynamics and disease.

## 5 CONCLUSION

In summary, we report the use of a comprehensive mitochondrial phenotype analysis (MitoCellPhe) tool that can identify in detail the presence of disparate mitochondrial morphologies in LS‐hiPSCs and fibroblasts that contain different mtDNA variants. Our observations allowed a detailed analysis of mitochondrial morphologies that is of utmost importance for studying the effect of the specific mtDNA variants. The results obtained indicate that specific point mutations in the mtDNA could contribute to subtle alterations in mitochondrial phenotypes that are variant‐specific. Overall, this study expands our knowledge of the variable morphologies adapted by mitochondria in patient‐ and disease‐specific hiPSCs and could lead to further clarification of the correlation between mitochondrial dynamics, genetics, and bioenergetics, and contribute to better diagnosis and specific therapies for the treatment of devastating mitochondrial disorders like LS.

It is important to note that hiPSCs prefer to grow in colonies, which contributes to complexities in the normalization process. In this study, we normalized the data to the cell number, which was variable in certain lines. The percentage that was unaffected by the cell density can be used as a definite parameter, or future normalization to cell number and surface area can be followed; however, it may be more time and labor‐intensive. In addition, the initial image quality is essential for the software's proper functioning. Nevertheless, this quantitative analysis permitted us to identify distinctions in mitochondrial dynamics in hiPSCs derived from patient fibroblasts that exhibit specific mtDNA mutations. In the long term, we hope that a thorough assessment of mitochondrial dynamics, along with correlations with genetic and bioenergetics analyses in hiPSCs, will aid in identifying the “signature” of complex mitochondrial disorders during early development.

## AUTHOR CONTRIBUTIONS

**Fibi Meshrkey:** Data curation; formal analysis; investigation; validation; visualization. **Ajibola B. Bakare:** Data curation; formal analysis; investigation. **Raj R. Rao:** Formal analysis; investigation. **Shilpa Iyer:** Conceptualization; formal analysis; funding acquisition; investigation; methodology; resources; supervision; visualization.

## FUNDING INFORMATION

This research was supported in part by funding from DoD W81XWH‐16‐1‐0181 and NIH 5R01HD110536‐03 (S. Iyer).

## CONFLICT OF INTEREST STATEMENT

The authors declare no conflicts of interest.

## ETHICS STATEMENT

The current study was conducted with the approval of the University Of Arkansas Office Of Research Compliance, which determined that the project was exempt from Institutional Review Board (IRB) oversight and human research subjects protection regulations.

## Supporting information


**Figure S1.** Mitochondrial morphology of healthy control (CTL) BJ‐FB and SBG2‐(*T8993G*)‐FB in the absence and presence of FCCP. Figures showing (A) total network branch count, (B) all branch count, (C) punctate%, (D) rod%, (E) network% %, (F) mean network branch length, and (G) mean network and rod length. All data are representative of 10–14 images taken from three independent dishes per treatment group. The bars represent minimum and maximum values, and each black dot represents a different data point. The dark and light gray bars represent the control fibroblast without and with FCCP treatment (−FCCP vs. +FCCP). The red and pink bars represent the SBG2‐(*T8993G*)‐FB without and with FCCP treatment (−FCCP vs. +FCCP).

**Figure S2.** Mitochondrial morphology of healthy control (CTL) BJ‐FB and SBG3‐(*T9185C*)‐FB in the absence and presence of FCCP. Figures showing (A) total network branch count, (B) all branch count, (C) punctate%, (D) rod%, (E) network% %, (F) mean network branch length, and (G) mean network and rod length. All data are representative of 10–14 images taken from three independent dishes per treatment group. The bars represent minimum and maximum values, and each black dot represents a different data point. The dark and light gray bars represent the control fibroblast without and with FCCP treatment (−FCCP vs. +FCCP). The red and pink bars represent the SBG3‐(*T9185C*)‐FB without and with FCCP treatment (−FCCP vs. +FCCP).

**Figure S3.** Mitochondrial morphology of healthy control (CTL) BJ‐FB and SBG4‐(*T10158C*)‐FB in the absence and presence of FCCP. Figures showing (A) total network branch count, (B) all branch count, (C) punctate%, (D) rod%, (E) network% %, (F) mean network branch length, and (G) mean network and rod length. All data are representative of 10–14 images taken from three independent dishes per treatment group. The bars represent minimum and maximum values, and each black dot represents a different data point. The dark and light gray bars represent the control fibroblast without and with FCCP treatment (−FCCP vs. +FCCP). The blue and green bars represent the SBG4‐(*T10158C*)‐FB without and with FCCP treatment (‐FCCP vs. +FCCP).

**Figure S4.** Mitochondrial morphology of healthy control (CTL) BJ‐FB and SBG5‐(*T12706C*)‐FB in the absence and presence of FCCP. Figures showing (A) total network branch count, (B) all branch count, (C) punctate%, (D) rod%, (E) network% %, (F) mean network branch length, and (G) mean network and rod length. All data are representative of 10–14 images taken from three independent dishes per treatment group. The bars represent minimum and maximum values, and each black dot represents a different data point. The dark and light gray bars represent the control fibroblast without and with FCCP treatment (−FCCP vs. +FCCP). The blue and green bars represent the SBG5‐(*T12706C*)‐FB without and with FCCP treatment (−FCCP vs. +FCCP).

**Figure S5.** Mitochondrial morphology of SBG2‐(*T8993G*) hiPSC in comparison to healthy control BJ‐hiPSC. Different mitochondrial morphological parameters were determined and analyzed in comparison with the BJ‐hiPSC (control cell line) to quantify (A) total network branch count, (B) all branch count, (C) punctate percentage (D) rods percentage (E) network percentage (F) mean network branches length and (G) mean network and rod length. All data are representative of five to seven analyzed images obtained from seven to nine independent dishes from three independent experiments. The bars represent minimum and maximum values, including all points, and each black dot represents a different data point. The gray bars represent the BJ‐control hiPSC, whereas the pink bars represent SBG2‐(*T8993G*)‐hiPSC.

**Figure S6.** Mitochondrial morphology of SBG3‐(*T9185C*)‐hiPSC in comparison to healthy control BJ‐hiPSC. Different mitochondrial morphological parameters were determined and analyzed in comparison with the BJ‐hiPSC (control cell line) to quantify (A) total network branch count, (B) all branch count, (C) punctate percentage (d) rods percentage (E) network percentage (F) mean network branches length and (G) mean network and rod length. All data are representative of five to seven analyzed images obtained from seven to nine independent dishes from three independent experiments. The bars represent minimum and maximum values, including all points, and each black dot represents a different data point. The gray bars represent the BJ‐control hiPSC, whereas the pink bars represent the SBG3‐(*T9185C*)‐hiPSC.

**Figure S7.** Mitochondrial morphology of SBG4‐(*T10158C*)‐hiPSC in comparison to healthy control BJ‐hiPSC. Different mitochondrial morphological parameters were determined and analyzed in comparison with the BJ‐hiPSC (control cell line) to quantify (A) total network branch count (B) all branch count (C) punctate percentage (D) rods percentage (E) network percentage (F) mean network branches length, and (G) mean network and rod length. All data are representative of five to seven analyzed images obtained from seven to nine independent dishes from three independent experiments. The bars represent minimum and maximum values, including all points, and each black dot represents a different data point. The gray bars represent the BJ‐control hiPSC, whereas the blue bars represent the SBG4‐(*T10158C*)‐hiPSC.

**Figure S8.** Mitochondrial morphology of SBG5‐(*T12706C*)‐hiPSC in comparison to healthy control BJ‐hiPSC. Different mitochondrial morphological parameters were determined and analyzed in comparison with the BJ‐hiPSC (control cell line) to quantify (A) total network branch count, (B) all branch count, (C) punctate percentage (D) rods percentage (E) network percentage (F) mean network branches length, and (G) mean network and rod length. All data are representative of five to seven analyzed images obtained from seven to nine independent dishes from three independent experiments. The bars represent minimum and maximum values, including all points, and each black dot represents a different data point. The gray bars represent the BJ‐control hiPSC, whereas the blue bars represent the SBG5‐(*T12706C*)‐hiPSC.

**Figure S9.** Representative images of BJ cells. Phase contrast, Mitotracker red, Nucblue, and Skeletonized images of BJ‐FBs with and without FCCP (A) and of BJ‐hiPSC (B) are demonstrated. Scale bar = 100 μm.

**Table S1.** Explanation of the different parameters obtained from MitoCellPhe. The different values generated by the MitoCellPhe analyzer and the definitions are detailed below.

## Data Availability

All the data supporting the results can be found in the manuscript and the supplemental data. No dataset has been deposited in a repository, and the data from the studied patient fibroblasts are not publicly available, in agreement with privacy law and our institutional policies. Please contact the corresponding author if materials generated from the current study are reasonably required.
