# Supplementary figures and images for: Mitochondrial morphology in human fibroblasts and induced pluripotent stem cells in Leigh syndrome: A comparative analysis
Source: Physiol Rep. 2026 May 10;14(9):e70911. doi: 10.14814/phy2.70911 (PMC13158366; doi:10.14814/phy2.70911)

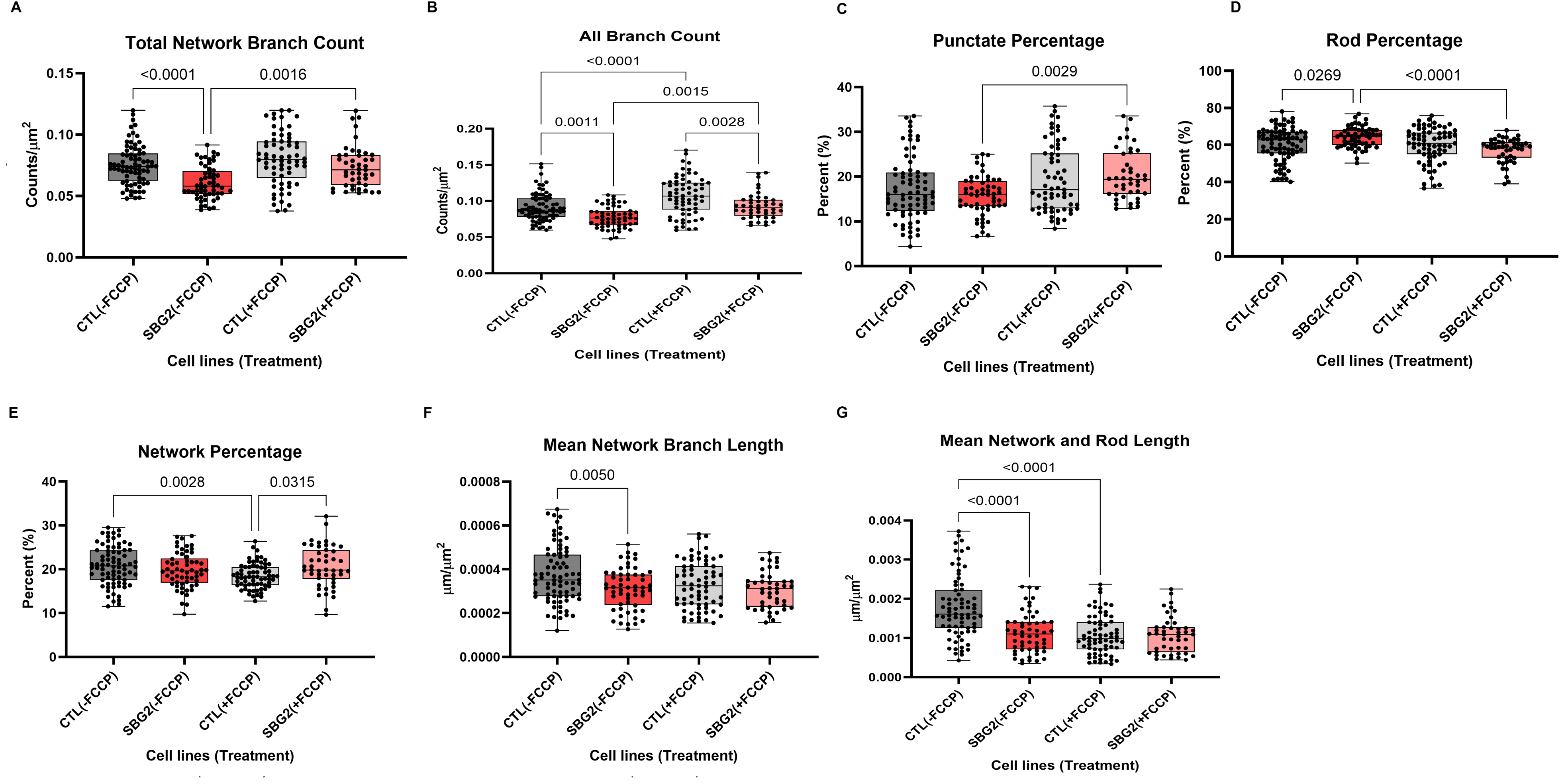

Supplement: Supplementary file 1 — Figure S1. Mitochondrial morphology of healthy control (CTL) BJ‐FB and SBG2‐(T8993G)‐FB in the absence and presence of FCCP. Figures showing (A) total network branch count, (B) all branch count, (C) punctate%, (D) rod%, (E) network% %, (F) mean network branch length, and (G) mean network and rod length. All data are representative of 10–14 images taken from three independent dishes per treatment group. The bars represent minimum and maximum values, and each black dot represents a different data point. The dark and light gray bars represent the control fibroblast without and with FCCP treatment (−FCCP vs. +FCCP). The red and pink bars represent the SBG2‐(T8993G)‐FB without and with FCCP treatment (−FCCP vs. +FCCP). [file PHY2-14-e70911-s003.tif]

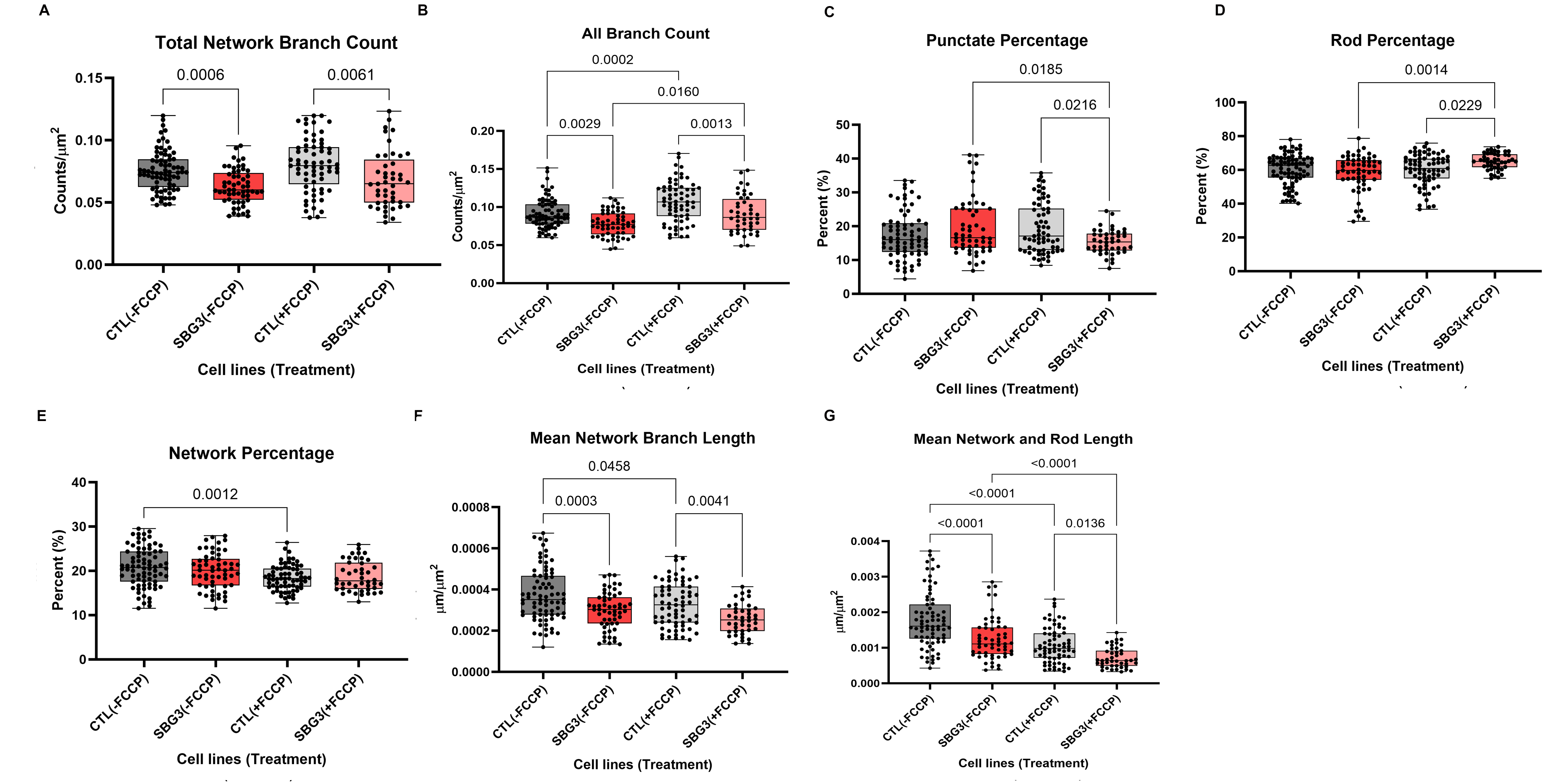

Supplement: Supplementary file 2 — Figure S2. Mitochondrial morphology of healthy control (CTL) BJ‐FB and SBG3‐(T9185C)‐FB in the absence and presence of FCCP. Figures showing (A) total network branch count, (B) all branch count, (C) punctate%, (D) rod%, (E) network% %, (F) mean network branch length, and (G) mean network and rod length. All data are representative of 10–14 images taken from three independent dishes per treatment group. The bars represent minimum and maximum values, and each black dot represents a different data point. The dark and light gray bars represent the control fibroblast without and with FCCP treatment (−FCCP vs. +FCCP). The red and pink bars represent the SBG3‐(T9185C)‐FB without and with FCCP treatment (−FCCP vs. +FCCP). [file PHY2-14-e70911-s005.tif]

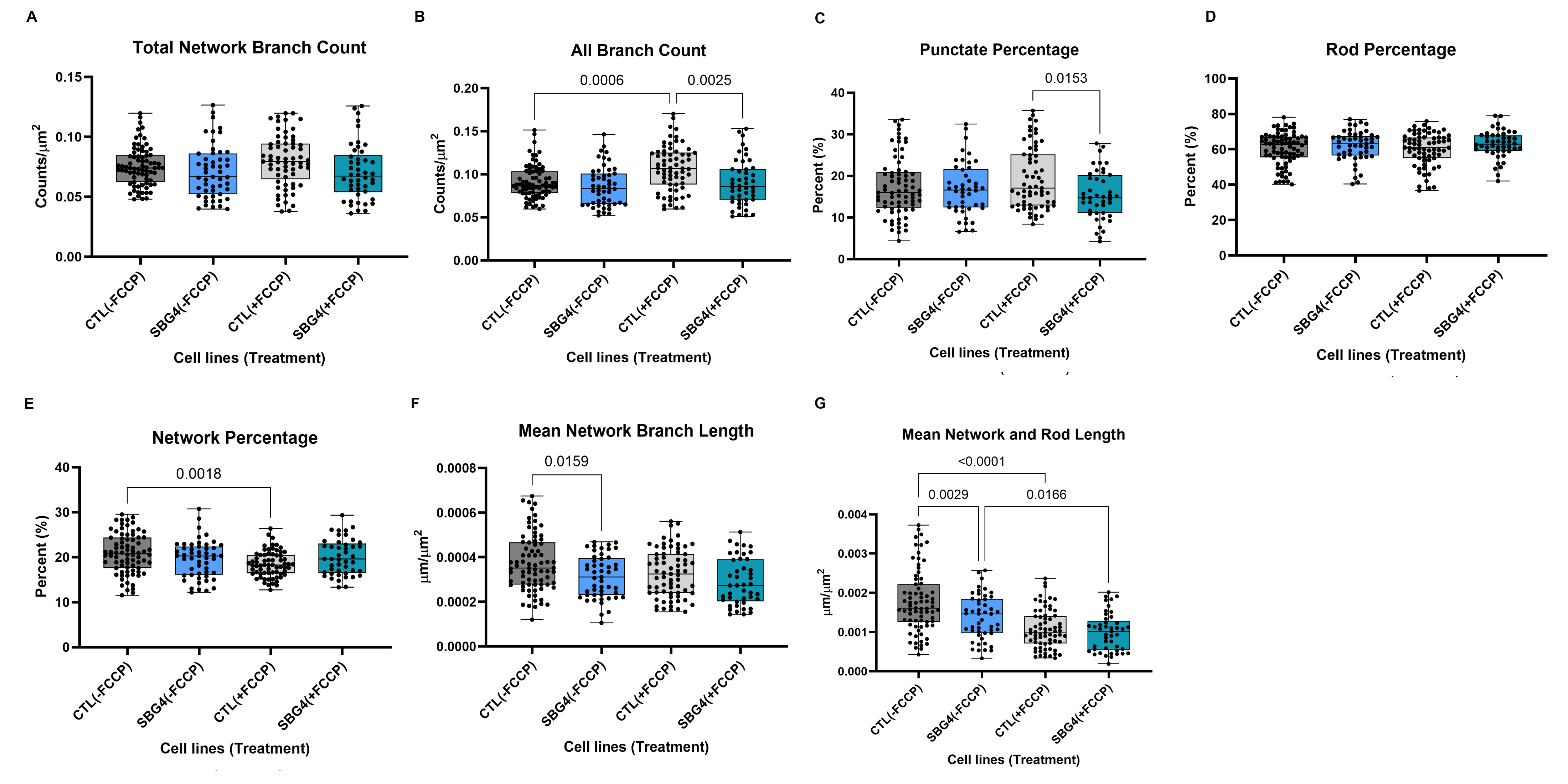

Supplement: Supplementary file 3 — Figure S3. Mitochondrial morphology of healthy control (CTL) BJ‐FB and SBG4‐(T10158C)‐FB in the absence and presence of FCCP. Figures showing (A) total network branch count, (B) all branch count, (C) punctate%, (D) rod%, (E) network% %, (F) mean network branch length, and (G) mean network and rod length. All data are representative of 10–14 images taken from three independent dishes per treatment group. The bars represent minimum and maximum values, and each black dot represents a different data point. The dark and light gray bars represent the control fibroblast without and with FCCP treatment (−FCCP vs. +FCCP). The blue and green bars represent the SBG4‐(T10158C)‐FB without and with FCCP treatment (‐FCCP vs. +FCCP). [file PHY2-14-e70911-s001.tif]

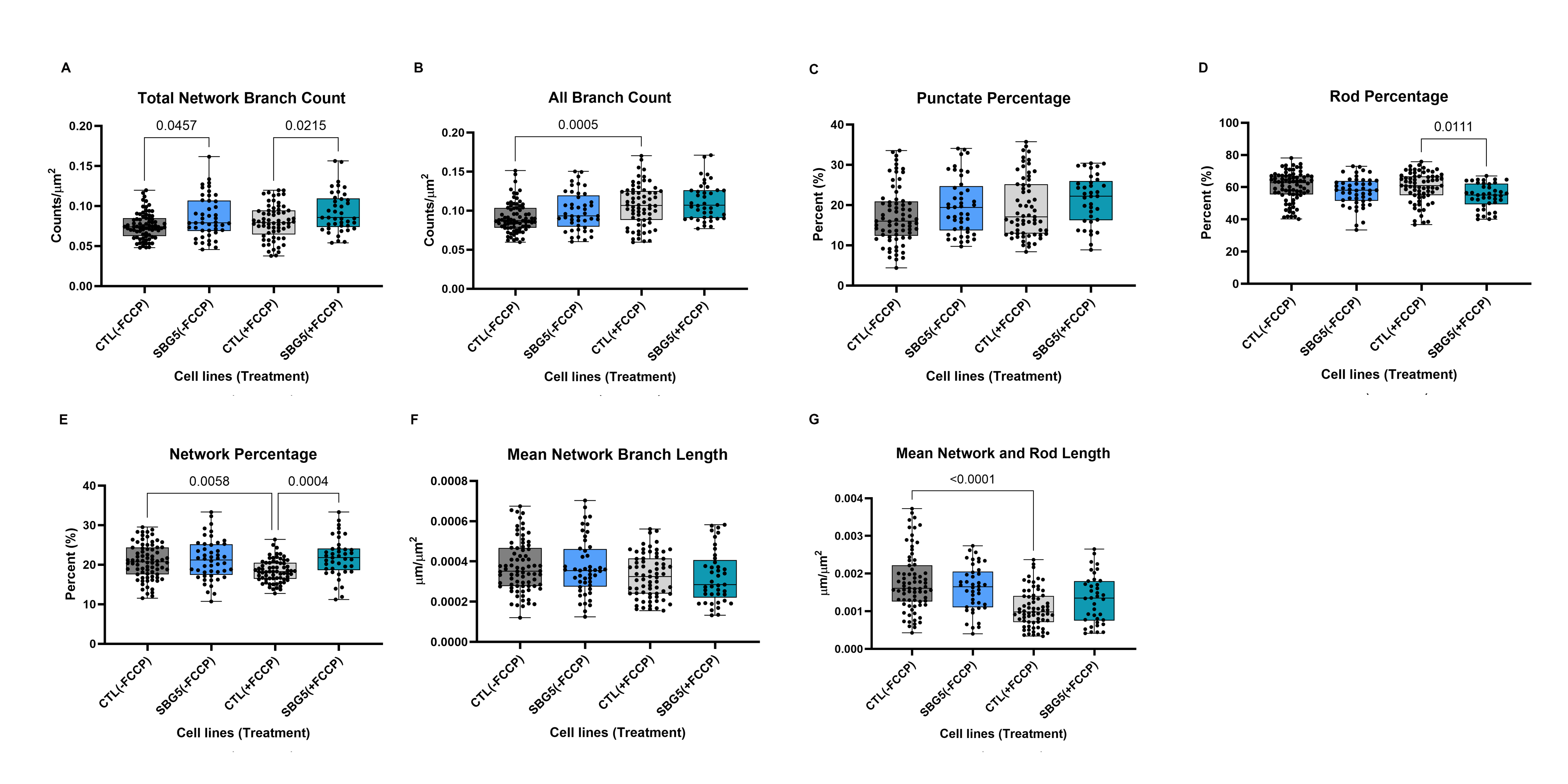

Supplement: Supplementary file 4 — Figure S4. Mitochondrial morphology of healthy control (CTL) BJ‐FB and SBG5‐(T12706C)‐FB in the absence and presence of FCCP. Figures showing (A) total network branch count, (B) all branch count, (C) punctate%, (D) rod%, (E) network% %, (F) mean network branch length, and (G) mean network and rod length. All data are representative of 10–14 images taken from three independent dishes per treatment group. The bars represent minimum and maximum values, and each black dot represents a different data point. The dark and light gray bars represent the control fibroblast without and with FCCP treatment (−FCCP vs. +FCCP). The blue and green bars represent the SBG5‐(T12706C)‐FB without and with FCCP treatment (−FCCP vs. +FCCP). [file PHY2-14-e70911-s006.tif]

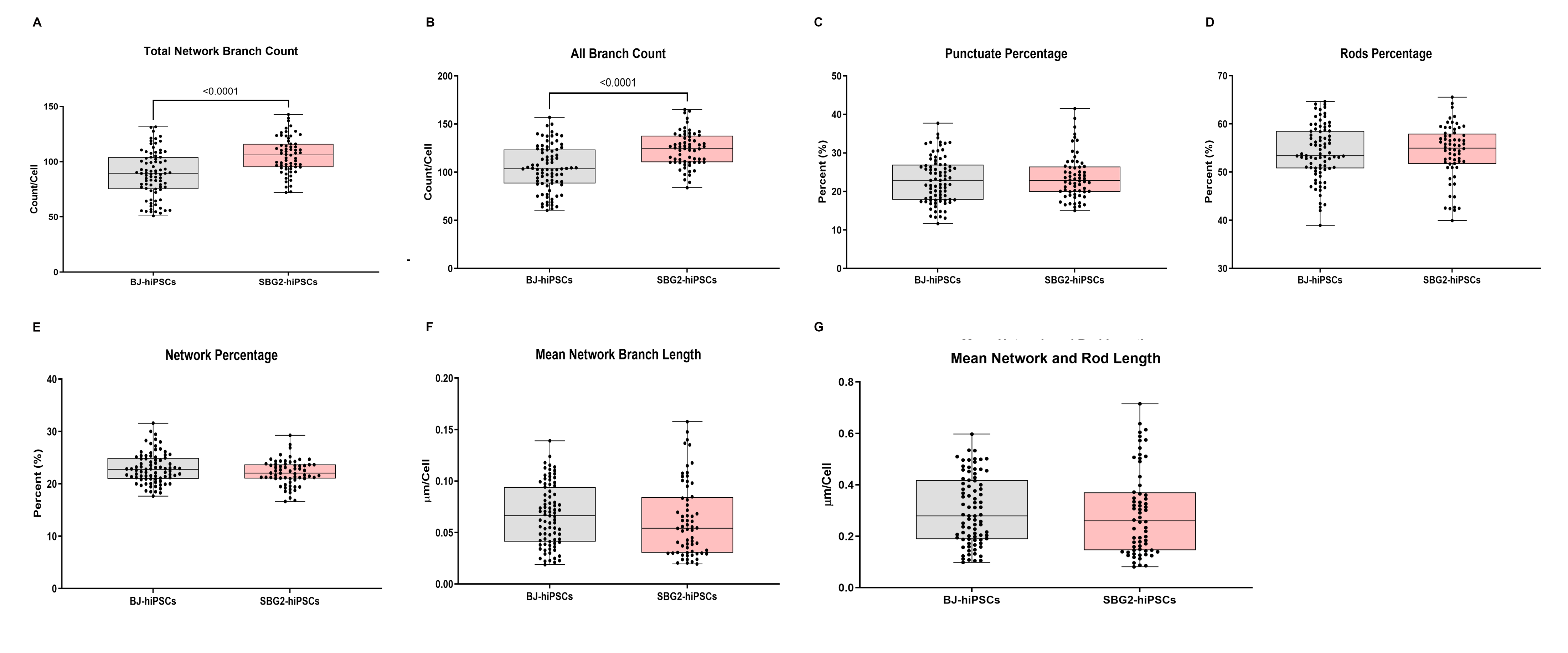

Supplement: Supplementary file 5 — Figure S5. Mitochondrial morphology of SBG2‐(T8993G) hiPSC in comparison to healthy control BJ‐hiPSC. Different mitochondrial morphological parameters were determined and analyzed in comparison with the BJ‐hiPSC (control cell line) to quantify (A) total network branch count, (B) all branch count, (C) punctate percentage (D) rods percentage (E) network percentage (F) mean network branches length and (G) mean network and rod length. All data are representative of five to seven analyzed images obtained from seven to nine independent dishes from three independent experiments. The bars represent minimum and maximum values, including all points, and each black dot represents a different data point. The gray bars represent the BJ‐control hiPSC, whereas the pink bars represent SBG2‐(T8993G)‐hiPSC. [file PHY2-14-e70911-s002.tif]

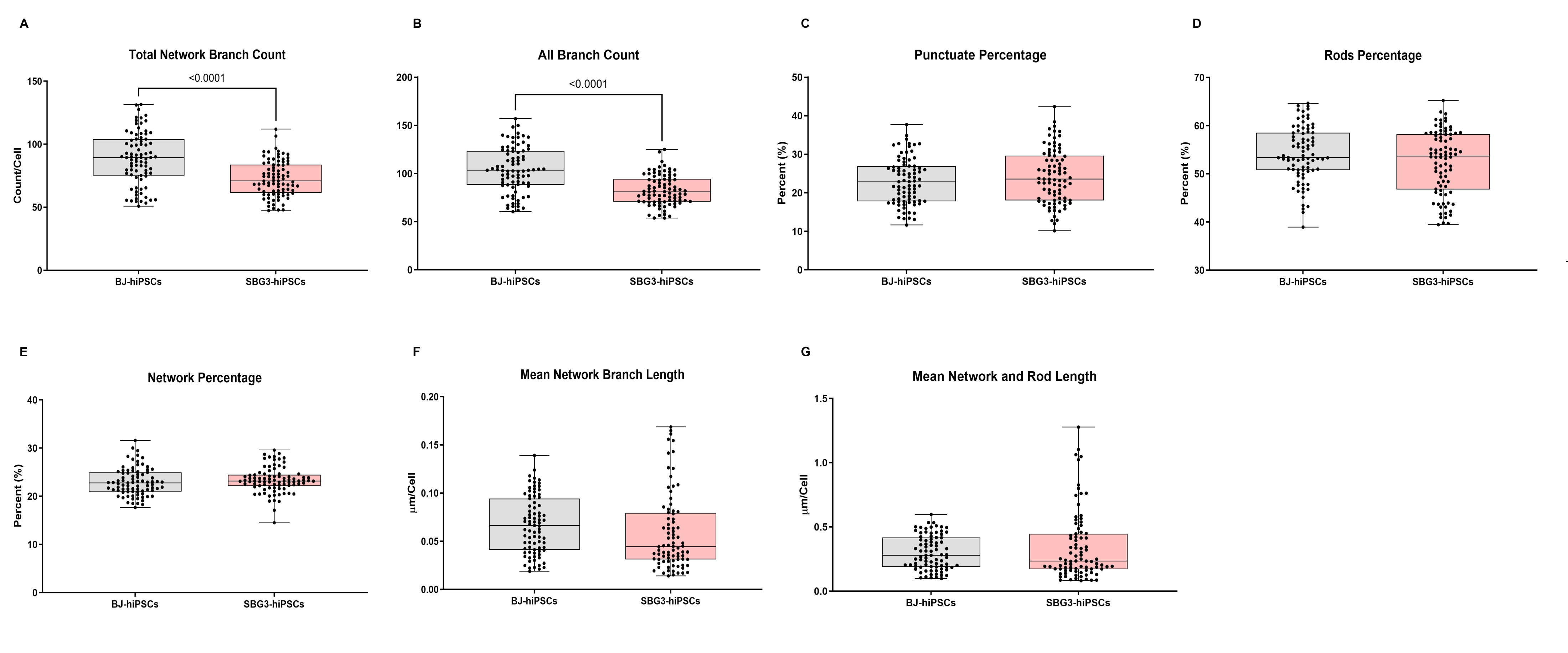

Supplement: Supplementary file 6 — Figure S6. Mitochondrial morphology of SBG3‐(T9185C)‐hiPSC in comparison to healthy control BJ‐hiPSC. Different mitochondrial morphological parameters were determined and analyzed in comparison with the BJ‐hiPSC (control cell line) to quantify (A) total network branch count, (B) all branch count, (C) punctate percentage (d) rods percentage (E) network percentage (F) mean network branches length and (G) mean network and rod length. All data are representative of five to seven analyzed images obtained from seven to nine independent dishes from three independent experiments. The bars represent minimum and maximum values, including all points, and each black dot represents a different data point. The gray bars represent the BJ‐control hiPSC, whereas the pink bars represent the SBG3‐(T9185C)‐hiPSC. [file PHY2-14-e70911-s010.tif]

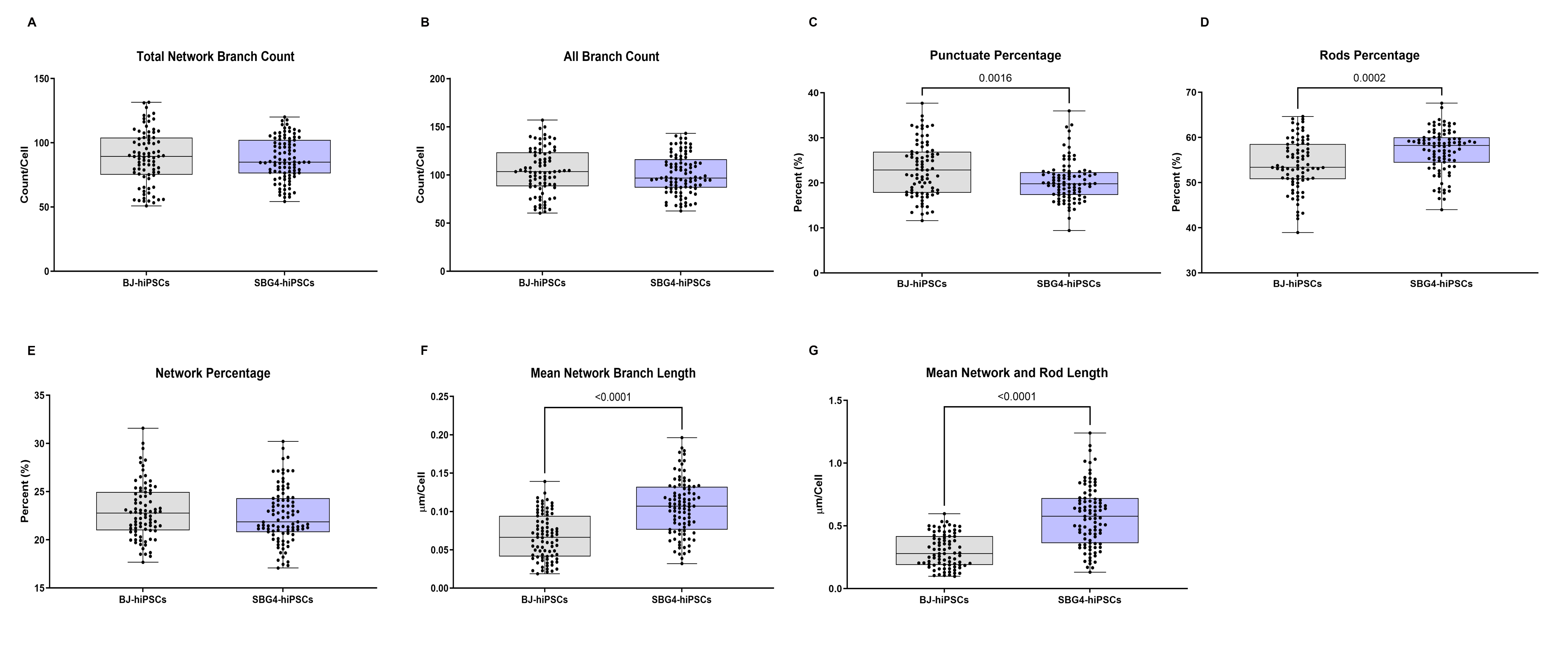

Supplement: Supplementary file 7 — Figure S7. Mitochondrial morphology of SBG4‐(T10158C)‐hiPSC in comparison to healthy control BJ‐hiPSC. Different mitochondrial morphological parameters were determined and analyzed in comparison with the BJ‐hiPSC (control cell line) to quantify (A) total network branch count (B) all branch count (C) punctate percentage (D) rods percentage (E) network percentage (F) mean network branches length, and (G) mean network and rod length. All data are representative of five to seven analyzed images obtained from seven to nine independent dishes from three independent experiments. The bars represent minimum and maximum values, including all points, and each black dot represents a different data point. The gray bars represent the BJ‐control hiPSC, whereas the blue bars represent the SBG4‐(T10158C)‐hiPSC. [file PHY2-14-e70911-s004.tif]

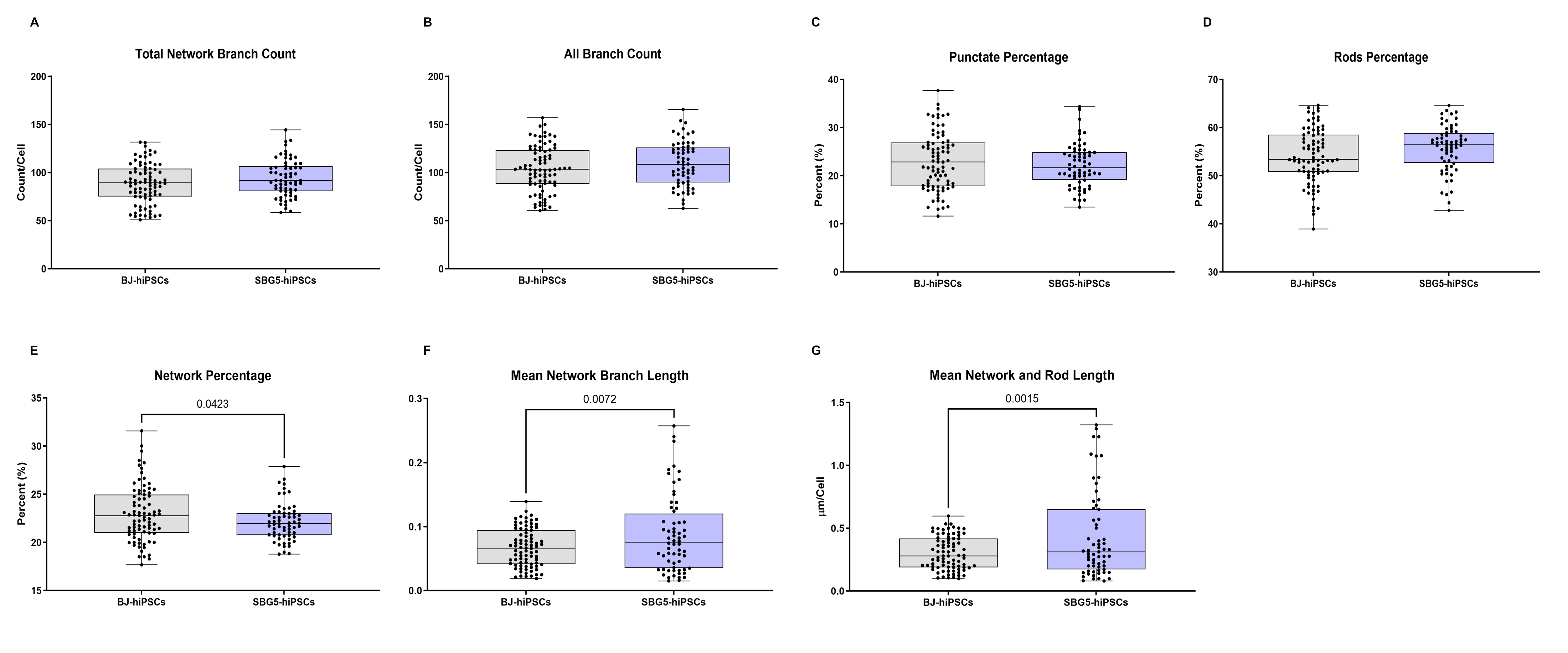

Supplement: Supplementary file 8 — Figure S8. Mitochondrial morphology of SBG5‐(T12706C)‐hiPSC in comparison to healthy control BJ‐hiPSC. Different mitochondrial morphological parameters were determined and analyzed in comparison with the BJ‐hiPSC (control cell line) to quantify (A) total network branch count, (B) all branch count, (C) punctate percentage (D) rods percentage (E) network percentage (F) mean network branches length, and (G) mean network and rod length. All data are representative of five to seven analyzed images obtained from seven to nine independent dishes from three independent experiments. The bars represent minimum and maximum values, including all points, and each black dot represents a different data point. The gray bars represent the BJ‐control hiPSC, whereas the blue bars represent the SBG5‐(T12706C)‐hiPSC. [file PHY2-14-e70911-s007.tif]

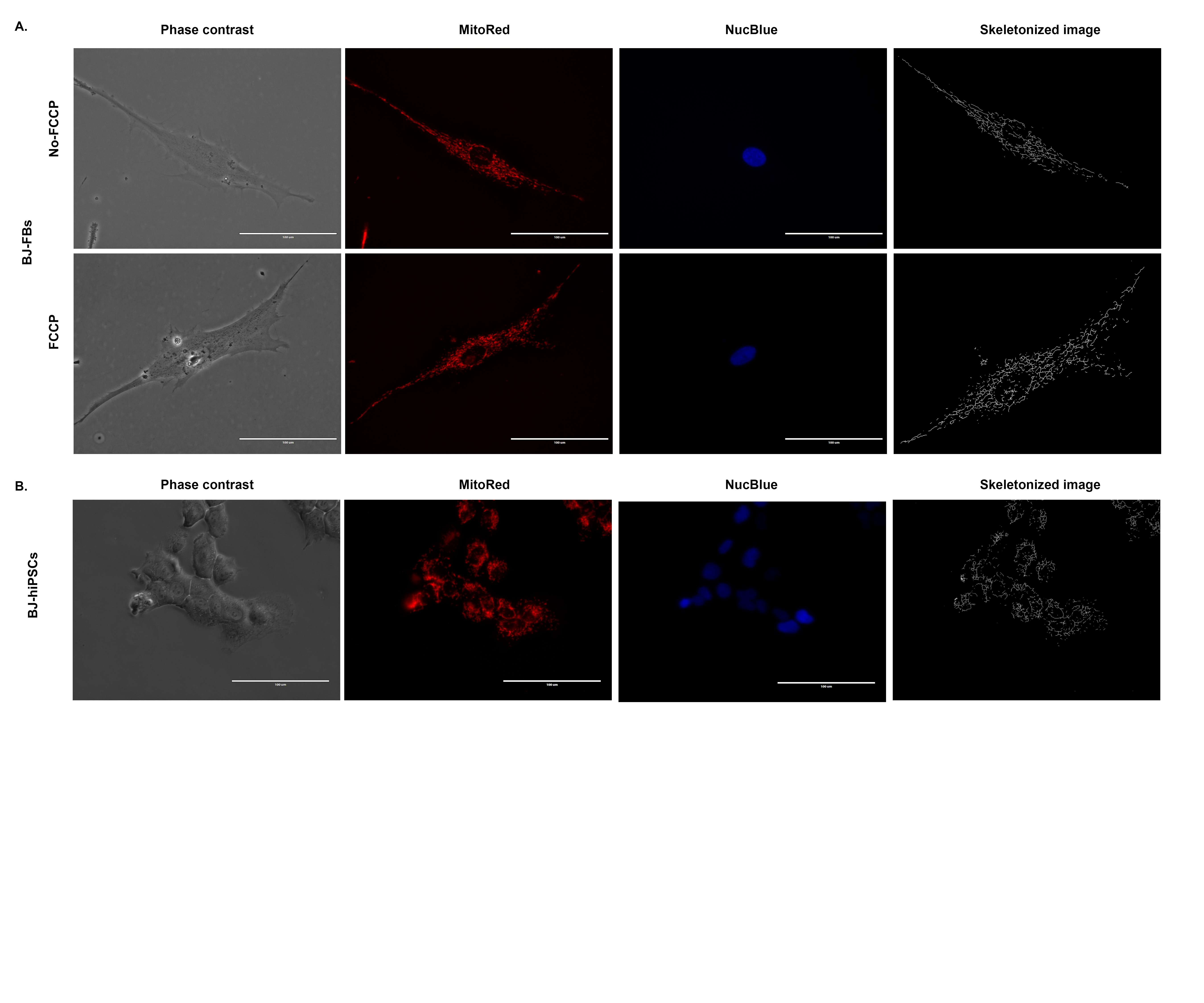

Supplement: Supplementary file 9 — Figure S9. Representative images of BJ cells. Phase contrast, Mitotracker red, Nucblue, and Skeletonized images of BJ‐FBs with and without FCCP (A) and of BJ‐hiPSC (B) are demonstrated. Scale bar = 100 μm. [file PHY2-14-e70911-s009.tif]
