# Supplementary material for: Mitochondrial morphology in human fibroblasts and induced pluripotent stem cells in Leigh syndrome: A comparative analysis
Source: Physiol Rep. 2026 May 10;14(9):e70911. doi: 10.14814/phy2.70911 (PMC13158366; doi:10.14814/phy2.70911)
Supplement: Supplementary file 10 — Table S1. Explanation of the different parameters obtained from MitoCellPhe. The different values generated by the MitoCellPhe analyzer and the definitions are detailed below. [file PHY2-14-e70911-s008.docx]

**Supplementary Table 1. Explanation of the different parameters obtained from MitoCellPhe.** The different values generated by the MitoCellPhe analyzer and the definitions are detailed below.

| ***Value*** | ***Definition*** |
| --- | --- |
| Skeleton Area | The area of the skeleton within the image (i.e., the number of pixels the skeleton takes, converted to micrometers) |
| Punctate Count | Number of skeletal components with zero branches (i.e., single points) |
| Rod Count | Number of skeletal components with one branch (i.e., straight lines) |
| Network Count | Number of skeletal segments with two or more branches |
| Punctate Percentage | The ratio of punctate components to the total number of components |
| Rod Percentage | The ratio of rod components to the total number of components |
| Network Percentage | The ratio of network components to the total number of components |
| Punctate Length | Always zero |
| Rod Length | The length of the branch of a rod |
| Network Length | The sum of all branch lengths contained within a network |
| Mean Rod Length | The average length of one-branch skeletal segments |
| Median Rod Length | The median length of one-branch skeletal segments |
| Stdev Rod Length | Population standard deviation of one-branch skeletal segments |
| Total Network Branch Count | Number of branches contained in all networks |
| Mean Network Branch Count | The average number of branches for each network |
| Mean Network Branch Length | Average branch length for each branch in every network |
| Mean Network Length | Average of every network’s length in an image |
| Median Network Length | Median of every network’s length in an image |
| Stdev Network Length | The population standard deviation of every network’s length in an image |
| All Branch Count | Number of branches contained in networks or rods |
| Mean Length of All Branches | Average branch length for each branch in every network and rod |
| Median Length of All Branches | Median branch length for every branch in every network and rod |
| Stdev Length of All Branches | Population standard deviation of the branch length for every branch in every network and rod |
| Mean Network and Rod Length | The average length of all networks and rods |
| Median Network and Rod Length | The median length of all networks and rods |
| Stdev Network and Rod Length | Population standard deviation of the length of all networks and rods |
